# Comparison of Clinical Characteristics Between Clinical Trial Participants and Nonparticipants Using Electronic Health Record Data

**DOI:** 10.1001/jamanetworkopen.2021.4732

**Published:** 2021-04-07

**Authors:** James R. Rogers, Cong Liu, George Hripcsak, Ying Kuen Cheung, Chunhua Weng

**Affiliations:** 1 Department of Biomedical Informatics, Columbia University, New York, New York; 2 Medical Informatics Services, New York–Presbyterian Hospital, New York, New York; 3 Department of Biostatistics, Columbia University, New York, New York

## Abstract

**Importance:**

Assessing generalizability of clinical trials is important to ensure appropriate application of interventions, but most assessments provide minimal granularity on comparisons of clinical characteristics.

**Objective:**

To assess the extent of underlying clinical differences between clinical trial participants and nonparticipants by using a combination of electronic health record and trial enrollment data.

**Design, Setting, and Participants:**

This cross-sectional study used data obtained from a single academic medical center between September 1996 and January 2019 to identify 1645 clinical trial participants from a diverse set of 202 available trials conducted at the center. Using an aggregated resampling procedure, nonparticipants were matched to participants 1:1 based on trial conditions, number of recent visits to a health care professional, and calendar time.

**Exposures:**

Clinical trial enrollment vs no enrollment.

**Main Outcomes and Measures:**

The primary outcome was standardized differences in clinical characteristics between participants and nonparticipants in clinical trials stratified into the 4 most common disease domains.

**Results:**

This cross-sectional study included 1645 participants from 202 trials (929 [56.5%] male; mean [SD] age, 54.65 [21.38] years) and an aggregated set of 1645 nonparticipants (855 [52.0%] male; mean [SD] age, 57.24 [21.91] years). The most common disease domains for the selected trials were neoplastic disease (86 trials; 737 participants), disorders of the digestive system (31 trials; 321 participants), inflammatory disorders (28 trials; 276 participants), and disorders of the cardiovascular system (27 trials; 319 participants); trials could qualify for multiple disease domains. Among 31 conditions, the percentage of conditions for which the prevalence was lower among participants than among nonparticipants per standardized differences was 64.5% (20 conditions) for neoplastic disease trials, 61.3% (19) for digestive system trials, 58.1% (18) for inflammatory disorder trials, and 38.7% (12) for cardiovascular system trials. Among 17 medications, the percentage of medications for which use was less among participants than among nonparticipants per standardized differences was 64.7% (11) for neoplastic disease trials, 58.8% (10) for digestive system trials, 88.2% (15) for inflammatory disorder trials, and 52.9% (9) for cardiovascular system trials.

**Conclusions and Relevance:**

Using a combination of electronic health record and trial enrollment data, this study found that clinical trial participants had fewer comorbidities and less use of medication than nonparticipants across a variety of disease domains. Combining trial enrollment data with electronic health record data may be useful for better understanding of the generalizability of trial results.

## Key Points

**Question:**

Are there differences in clinical characteristics between clinical trial participants and nonparticipants as captured by electronic health record data?

**Findings:**

In this cross-sectional study of 1645 clinical trial participants and an aggregated set of 1645 matched nonparticipants, most of the trial participants had fewer underlying conditions and less medication use than nonparticipants.

**Meaning:**

These findings suggest that a more comprehensive approach to evaluating trials may be beneficial for addressing concerns about the generalizability of clinical trial results.

## Teaser

This cross-sectional study uses electronic health records and clinical trial enrollment data to assess differences in the clinical characteristics of trial participants and nonparticipants.

## Introduction

Clinical trials are considered one of the best study designs for generating medical evidence. A common challenge for individuals interpreting clinical trials is assessing generalizability, which is the practice of determining how reasonably relevant the results are to a particular group of individuals who were not part of the trial.^1^ A variety of these comparisons have shown disparities across many disease domains. In cancer trials, many studies^2,3,4,5^ have noted that trial participants tend to be younger, with more promising prognoses and fewer comorbidities. In cardiovascular (CV) disease trials, participants are more likely to be male, with less risk for developing CV outcomes.^3,6^ In a meta-analysis of end-stage kidney disease trials, participants were found to be younger and to have different comorbidity profiles.^7^ Other disease domains in which similar concerns are noted include mental health, psoriasis, and type 2 diabetes.^3,8,9,10^ Understanding differences between trial participants and nonparticipants is important because underlying characteristics can influence the estimated effect of an intervention, ultimately impacting its clinical meaningfulness.^1^ However, many of these prior assessments examined only aggregated estimates between trial participants and nonparticipants with comparisons based on tabular data reported by the selected trials, providing limited insight when comparing the 2 groups. A novel combination of prior trial enrollment data and electronic health records (EHRs) may provide more granular and detailed assessments by leveraging individual participants’ medical history. To our knowledge, prior literature reviews on use of EHR data and other similar data sources for generalizability purposes have found no study exploring this particular linkage.^3,11,12^

The primary aim of this study was to compare clinical profiles of trial participants with those of nonparticipants as collected from their EHR profiles across different disease domains. We hypothesized that clinical differences would exist regardless of the disease domain. As a secondary aim, we examined associations between participant covariates and trial parameters (eg, randomization use and number of treatment arms). We hypothesized that some covariates would be associated with certain trial parameters, suggesting that certain covariates are evaluated only in certain types of trials.

## Methods

This cross-sectional study used data obtained from a single academic medical center between September 1996 and January 2019 to identify 1645 clinical trial participants from a diverse set of 202 available trials conducted at the center. The eFigure in the Supplement provides an overview of the methods. This study was approved by the Columbia University institutional review board and qualified for a waiver of informed consent per the Code of Federal Regulations (45 CFR 46.116). The study followed the Strengthening the Reporting of Observational Studies in Epidemiology (STROBE) reporting guideline.

### Data Sources

The study used 3 data sources. The first was EHR data from the Columbia University Irving Medical Center (CUIMC), an academic medical center in New York, New York. The database contains more than 4.5 million inpatient and outpatient records collected from October 1985 to March 2020. The data are stored in the format of the Observational Medical Outcomes Partnership common data model, version 05, developed and maintained by the Observational Health Data Science and Informatics collaborative.^13,14^ Data elements of interest were demographic characteristics, medical conditions, and medication prescriptions.

The second data source was an internal report on the participation status of 4022 individuals in 297 interventional medication trials that involved the CUIMC as either the primary site of the trial or a recruitment site for a multisite trial, with records collected from September 1996 to January 2019. The report only contains data regarding patients’ medical record numbers, trial identifiers, status (eg, randomized, completed), and dates of status. The data in the report and patients’ EHR data are linkable through patients’ medical record numbers. The CUIMC setting for these 2 data sources was conducive for the analysis because it is a large medical center in a dense metropolitan area, thus allowing for a large number of patients available, and it has a well-established research environment that supports numerous trials in tandem with clinical care.

The third data source used in this study was the Aggregate Analysis of ClinicalTrials.gov (AACT) database, a publicly available relational database containing records from ClinicalTrials.gov.^15^ The AACT database and the CUIMC internal report are linkable through trial identifiers. Data were extracted from the AACT database on May 13, 2020.

### Selection of Trial Participants

For each trial participant, we first identified the earliest status date, limiting each participant to their earliest trial. Based on the chosen date, each participant’s record was checked for at least 1 relevant condition code within the 365 days before and including the status date. A relevant condition code was defined as a condition of focus listed in the participant’s trial description per the AACT database. Extracted conditions were converted to standardized codes used in the Observational Medical Outcomes Partnership common data model; if all codes for a trial did not have available conversions, the trial and its accompanying patients were excluded. Likewise, if no code was found in a participant’s record, that participant was excluded. The code in the participant’s record had to be either a direct match or a descendant. The code closest to the status date was used to define the index date for that participant. As a reassurance requirement to increase the confidence of the participant having the index condition, each participant was also required to have the designated index condition or 1 of its descendants recorded as present 365 days before, but not including, the index date.

### Selection of Nonparticipants

To identify the pool of nonparticipants, candidates were identified based on the index condition codes used for the trial participants. The aforementioned reassurance requirement was also applied to each candidate. Each participant was then matched to 1 randomly selected nonparticipant based on (1) the index condition, meaning the same condition code; (2) the calendar month and year of the index to control for potential temporal biases in how the data were recorded; and (3) the number of visits to a health care professional within the 365 days before the index date, which was chosen to approximate health care use. Each nonparticipant could be matched to only 1 participant. This matching procedure was repeated 1000 times. If a participant could not be matched in all 1000 iterations, that participant was excluded. Although this procedure relied on straightforward variable-to-variable matching, we chose it over more sophisticated techniques such as propensity scores because the latter would potentially interfere with the study’s primary outcome. Specifically, involvement of clinical covariates for matching could minimize differences between the 2 groups, but these differences were precisely the primary focus of this study.

### Statistical Analysis

Descriptive statistics for trials, participants, and nonparticipants are presented. Trial characteristics were selected based on available study design information from the AACT database. Clinical characteristics (ie, covariates) were based on demographic characteristics, medical conditions, and medication history and were stratified by clinical trial disease domain. Disease domains were derived from ancestor codes for the trials’ condition(s) of focus; we focused on disease domains with the most trials.

All data analyses were performed using R statistical software, version 3.5.1 (R Project for Statistical Computing). Covariates were derived using the Observational Health Data Science and Informatics FeatureExtraction package, version 2.2.5, for R.^16^ We chose these covariates because they constitute a diverse clinical profile, including prior malignancies, CV diseases, medication prescriptions, and other underlying comorbidities. An individual qualified for a covariate if there was at least 1 code in the individual’s record within the 365 days before and including the index date (relevant code definitions are available on request). To establish descriptive statistics for nonparticipants, we used the mean of the 1000 estimates. Standardized differences were used for assessment because they provide a robust analysis for evaluating covariate imbalance between 2 groups and provide a streamlined approach to identifying covariates that differ most substantially; the cutoff to find differences was set at an absolute difference greater than or equal to 0.1.^17,18^

As a secondary analysis, we examined the association between trial parameters and participant covariates, stratified by disease domain. We performed χ^2^ (or Fisher exact) tests for each pairing, with the 2-tailed significance level set at *P* < .01. To account for multiple testing, we applied a Bonferroni correction within each disease domain. Manhattan-like plots were created to visualize results using the ggplot2 package, version 3.3.2, for R.^19^

## Results

### Trial Characteristics

After applying cohort requirements, a total of 202 trials with 1645 participants were available for analysis (eTable 1 in the Supplement); 929 (56.5%) were male, and the mean (SD) age was 54.65 (21.38) years. Of the aggregated set of 1645 nonparticipants, 855 (52.0%) were male, and the mean (SD) age was 57.24 (21.91) years (additional baseline information is available in eTable 2 in the Supplement). The most common disease domains were neoplastic disease (86 trials; 737 participants), disorders of the digestive system (31 trials; 321 participants), inflammatory disorders (28 trials; 276 participants), and disorders of the CV system (27 trials; 319 participants); trials could qualify for multiple disease domains, so the disease domains were not mutually exclusive. The most common disease in the neoplastic domain in terms of both the number of trials and the number of participants was lymphoma (22 trials; 146 patients). Hepatitis C virus was the most common disease among digestive system disorders and inflammatory disorders (17 trials and 146 patients in each disease domain). For disorders of the CV system, hypertensive disorder was the most common in terms of the number of trials (8), and myocardial disease was the most common in terms of the number of participants (77).

Table 1 summarizes the trials’ characteristics. The most common trial phase across all disease domains was phase 2 with the exception of CV system trials, which were mostly phase 3. Neoplastic disease was the only disease domain in which most trials did not mention the use of a randomization procedure, whereas the CV system domain had the highest proportion of trials that used a randomization procedure. Across all disease domains, the majority of trials were multisite, had an industry sponsor, involved a data monitoring committee, and recruited fewer than 20 patients at the institution.

**Table 1.** Trial Characteristics Stratified by Disease Domain

Characteristic^a^	Trials, No. (%)				
	Overall (N = 202)	Neoplastic disease (n = 86)	Disorders of digestive system (n = 31)	Inflammatory disorders (n = 28)	Disorders of cardiovascular system (n = 27)
Trial phase^b^					
Phase 1	28 (13.9)	21 (24.4)	3 (9.7)	0	1 (3.7)
Phase 2	79 (39.1)	43 (50.0)	17 (54.8)	13 (46.4)	6 (22.2)
Phase 3	77 (38.1)	21 (24.4)	10 (32.3)	12 (42.9)	15 (55.6)
Phase 4	18 (8.9)	1 (1.2)	1 (3.2)	3 (10.7)	5 (18.5)
Treatment arms, No.					
1	56 (27.7)	30 (34.9)	9 (29.0)	7 (25.0)	7 (25.9)
2	89 (44.1)	28 (32.6)	10 (32.3)	11 (39.3)	16 (59.3)
≥3	57 (28.2)	28 (32.6)	12 (38.7)	10 (35.7)	4 (14.8)
Any mentioned use of randomization	117 (57.9)	35 (40.7)	19 (61.3)	19 (67.9)	20 (74.1)
Any mentioned use of blinding	81 (40.1)	14 (16.3)	10 (32.3)	8 (28.6)	16 (59.3)
Intervention model					
Single group assignment	67 (33.2)	39 (45.3)	10 (32.3)	9 (32.1)	6 (22.2)
Parallel assignment	125 (61.9)	41 (47.7)	21 (67.7)	19 (67.9)	20 (74.1)
Other assignment method	10 (5.0)	6 (7.0)	0	0	1 (3.7)
Industry sponsor involvement	182 (90.1)	73 (84.9)	28 (90.3)	24 (85.7)	25 (92.6)
Use of a data monitoring committee	140 (69.3)	50 (58.1)	24 (77.4)	22 (78.6)	21 (77.8)
Multisite trial	169 (83.7)	73 (84.9)	27 (87.1)	24 (85.7)	23 (85.2)
Overall trial enrollment, No.^c^					
<50	49 (24.3)	24 (27.9)	7 (22.6)	5 (17.9)	4 (14.8)
50-499	101 (50.0)	46 (53.5)	17 (54.8)	15 (53.6)	9 (33.3)
≥500	52 (25.7)	16 (18.6)	7 (22.6)	8 (28.6)	14 (51.9)
Institution-specific enrollment, No.^d^					
<5	48 (23.8)	17 (19.8)	6 (19.4)	6 (21.4)	6 (22.2)
5-19	111 (55.0)	49 (57.0)	17 (54.8)	16 (57.1)	13 (48.1)
≥20	43 (21.3)	20 (23.3)	8 (25.8)	6 (21.4)	8 (29.6)
Status					
Completed	110 (54.5)	33 (38.4)	19 (61.3)	17 (60.7)	15 (55.6)
Recruiting	20 (9.9)	15 (17.4)	2 (6.5)	1 (3.6)	3 (11.1)
Active, not recruiting	40 (19.8)	28 (32.6)	3 (9.7)	3 (10.7)	2 (7.4)
Terminated, suspended, or withdrawn	25 (12.4)	8 (9.3)	5 (16.1)	6 (21.4)	5 (18.5)
Unknown status	7 (3.5)	2 (2.3)	2 (6.5)	1 (3.6)	2 (7.4)

^a^ All trial characteristic groups are mutually exclusive.

^b^ Trials listed as multiple or combination phases were analyzed as their latest phase (eg, phase 1/phase 2 was considered phase 2).

^c^ If actual enrollment was not available, anticipated enrollment was used.

^d^ Refers to the number of patients enrolled at Columbia University Irving Medical Center only, which was extracted from the available trial enrollment data.

### Comparison of Trial Participants and Nonparticipants

Table 2 and Table 3 provide covariate comparisons between trial participants and nonparticipants for each disease domain (nonstratified comparisons are shown in eTable 2 in the Supplement). For demographic covariates, substantial differences (ie, absolute value of the standardized difference ≥0.1) in age and race existed between trial participants and nonparticipants in digestive system trials, inflammatory disorder trials, and CV system trials. Differences between participants and nonparticipants in ethnicity were found for neoplastic trials, digestive system trials, and CV system trials. In addition, participants in digestive system trials and CV system trials were more likely to be male (201 of 321 participants [62.6%] in digestive system trials and 217 of 319 participants [68.0%] in CV system trials).

**Table 2.** Covariate Comparisons Between Participants and Nonparticipants in Trials for Neoplastic Disease and Digestive-System Disorders

Covariates	Trials, No. (%)					
	Neoplastic disease			Digestive-system disorders		
	Participants (n = 737)	Nonparticipants (n = 737)	Standardized difference	Participants (n = 321)	Nonparticipants (n = 321)	Standardized difference
Demographics						
Age group, y						
<18	69 (9.4)	52 (7.1)	0.080	0	20 (6.3)	−0.366^a^
18-64	331 (44.9)	322 (43.7)	0.025	239 (74.5)	197 (61.3)	0.284^b^
≥65	337 (45.7)	363 (49.2)	−0.069	82 (25.5)	104 (32.4)	−0.152^a^
Sex						
Male	391 (53.1)	378 (51.3)	0.035	201 (62.6)	185 (57.6)	0.100^b^
Female	346 (46.9)	359 (48.7)	−0.035	120 (37.4)	136 (42.4)	−0.100^a^
Ethnicity						
Hispanic or Latino	76 (10.3)	104 (14.1)	−0.117^a^	41 (12.8)	58 (17.9)	−0.143^a^
Not Hispanic or Latino	353 (47.9)	362 (49.1)	−0.025	153 (47.7)	147 (45.6)	0.041
Unknown	308 (41.8)	271 (36.7)	0.104^b^	127 (39.6)	117 (36.4)	0.065
Race						
White	353 (47.9)	339 (46.0)	0.037	177 (55.1)	142 (44.2)	0.220^b^
Black or African American	55 (7.5)	66 (9.0)	−0.057	14 (4.4)	33 (10.2)	−0.225^a^
Other^c^	28 (3.8)	31 (4.3)	−0.024	7 (2.2)	9 (2.9)	−0.048
Unknown	301 (40.8)	300 (40.7)	0.003	123 (38.3)	137 (42.7)	−0.089
Comorbidities						
Acute respiratory disease	50 (6.8)	71 (9.7)	−0.106^a^	22 (6.9)	36 (11.1)	−0.146^a^
Chronic liver disease	28 (3.8)	42 (5.6)	−0.087	164 (51.1)	162 (50.5)	0.012
Chronic obstructive lung disease	33 (4.5)	58 (7.9)	−0.141^a^	16 (5.0)	26 (8.2)	−0.129^a^
Depressive disorder	52 (7.1)	83 (11.3)	−0.145^a^	32 (10.0)	52 (16.3)	−0.186^a^
Diabetes	97 (13.2)	132 (17.9)	−0.131^a^	56 (17.4)	79 (24.6)	−0.178^a^
Gastroesophageal reflux disease	70 (9.5)	106 (14.4)	−0.151^a^	28 (8.7)	50 (15.7)	−0.215^a^
Hyperlipidemia	136 (18.5)	188 (25.5)	−0.171^a^	33 (10.3)	65 (20.3)	−0.280^a^
Hypertensive disorder	234 (31.8)	315 (42.7)	−0.227^a^	107 (33.3)	144 (45.0)	−0.241^a^
Lesion of liver	142 (19.3)	81 (11.0)	0.232^b^	164 (51.1)	131 (40.7)	0.210^b^
Obesity	23 (3.1)	43 (5.9)	−0.134^a^	13 (4.0)	29 (9.1)	−0.208^a^
Osteoarthritis	97 (13.2)	129 (17.6)	−0.121^a^	29 (9.0)	41 (12.8)	−0.122^a^
Pneumonia	52 (7.1)	90 (12.2)	−0.175^a^	10 (3.1)	29 (9.0)	−0.248^a^
Renal impairment	72 (9.8)	128 (17.3)	−0.221^a^	38 (11.8)	58 (18.1)	−0.177^a^
Urinary tract infectious diseases	54 (7.3)	93 (12.6)	−0.178^a^	9 (2.8)	24 (7.4)	−0.210^a^
Viral hepatitis C infection	11 (1.5)	19 (2.6)	−0.080	156 (48.6)	149 (46.6)	0.041
Visual system disorder	74 (10.0)	104 (14.1)	−0.125^a^	21 (6.5)	30 (9.3)	−0.103^a^
Atrial fibrillation	34 (4.6)	63 (8.6)	−0.162^a^	11 (3.4)	22 (6.8)	−0.154^a^
Cerebrovascular disease	31 (4.2)	45 (6.0)	−0.084	11 (3.4)	16 (5.0)	−0.081
Coronary arteriosclerosis	50 (6.8)	92 (12.4)	−0.192^a^	20 (6.2)	32 (10.1)	−0.141^a^
Heart disease	196 (26.6)	272 (36.9)	−0.223^a^	90 (28.0)	100 (31.3)	−0.072
Heart failure	30 (4.1)	64 (8.7)	−0.189^a^	15 (4.7)	27 (8.4)	−0.150^a^
Ischemic heart disease	13 (1.8)	41 (5.5)	−0.199^a^	4 (1.2)	19 (5.8)	−0.252^a^
Peripheral vascular disease	6 (0.8)	16 (2.2)	−0.113^a^	3 (0.9)	5 (1.7)	−0.070
Venous thrombosis	35 (4.7)	60 (8.1)	−0.141^a^	17 (5.3)	25 (7.9)	−0.105^a^
Hematologic neoplasm	320 (43.4)	316 (42.8)	0.012	16 (5.0)	20 (6.3)	−0.057
Malignant lymphoma	169 (22.9)	174 (23.6)	−0.016	2 (0.6)	7 (2.3)	−0.144^a^
Malignant neoplastic disease	737 (100.0)	736 (99.9)^d^	0.015	157 (48.9)	152 (47.5)	0.028
Malignant tumor of breast	101 (13.7)	121 (16.4)	−0.075	3 (0.9)	6 (1.8)	−0.078
Malignant tumor of lung	100 (13.6)	69 (9.4)	0.133^b^	21 (6.5)	15 (4.7)	0.077
Malignant tumor of urinary bladder	63 (8.5)	66 (8.9)	−0.015	2 (0.6)	0	0.110^b^
Primary malignant neoplasm of prostate	68 (9.2)	65 (8.8)	0.015	21 (6.5)	15 (4.8)	0.075
Medication use						
Agents acting on the renin-angiotensin system	171 (23.2)	201 (27.3)	−0.094	52 (16.2)	90 (28.0)	−0.288^a^
Antibacterial medications for systemic use	419 (56.9)	461 (62.5)	−0.115^a^	168 (52.3)	200 (62.2)	−0.200^a^
Antidepressant medications	146 (19.8)	152 (20.7)	−0.022	71 (22.1)	73 (22.9)	−0.019
Antiepileptic medications	160 (21.7)	172 (23.3)	−0.039	33 (10.3)	51 (16.0)	−0.170^a^
Anti-inflammatory and antirheumatic products	188 (25.5)	219 (29.7)	−0.093	89 (27.7)	118 (36.7)	−0.194^a^
Antineoplastic agents	273 (37.0)	341 (46.3)	−0.189^a^	61 (19.0)	69 (21.5)	−0.062
Antithrombotic agents	305 (41.4)	397 (53.9)	−0.251^a^	142 (44.2)	168 (52.4)	−0.165^a^
β blocking agents	159 (21.6)	205 (27.9)	−0.145^a^	87 (27.1)	109 (33.9)	−0.148^a^
Calcium channel blockers	133 (18.0)	168 (22.8)	−0.118^a^	71 (22.1)	68 (21.2)	0.021
Diuretic medications	176 (23.9)	219 (29.7)	−0.131^a^	91 (28.3)	112 (34.8)	−0.141^a^
Drugs for acid-related disorders	359 (48.7)	432 (58.6)	−0.200^a^	164 (51.1)	184 (57.3)	−0.125^a^
Drugs for obstructive airway diseases	137 (18.6)	175 (23.7)	−0.125^a^	39 (12.1)	75 (23.5)	−0.301^a^
Drugs used in diabetes	123 (16.7)	158 (21.4)	−0.121^a^	86 (26.8)	94 (29.4)	−0.057
Immunosuppressant medications	69 (9.4)	95 (12.9)	−0.111^a^	64 (19.9)	45 (13.9)	0.159^b^
Lipid-modifying agents	180 (24.4)	228 (31.0)	−0.148^a^	43 (13.4)	74 (23.1)	−0.252^a^
Opioids	395 (53.6)	409 (55.5)	−0.038	175 (54.5)	189 (58.8)	−0.088
Psycholeptic medications	358 (48.6)	361 (49.0)	−0.009	134 (41.7)	147 (45.7)	−0.081

^a^ Covariate was more prevalent among nonparticipants than among participants (ie, standardized difference ≤−0.1).

^b^ Covariate was more prevalent among participants than among nonparticipants (ie, standardized difference ≥0.1).

^c^ This category consists of any reported race that did not qualify for 1 of the other categories (ie, the reported race was not White, Black or African American, or Unknown).

^d^ This is not 100% because 1 record during resampling was matched to a participant based on a condition listed within the neoplastic disease trial, but that condition was not a neoplastic disease.

**Table 3.** Covariate Comparisons Between Participants and Nonparticipants in Trials for Inflammatory Disorders and Cardiovascular-System Disorders

Covariates	Trials, No. (%)					
	Inflammatory disorders			Cardiovascular-system disorders		
	Participants (n = 276)	Nonparticipants (n = 276)	Standardized difference	Participants (n = 319)	Nonparticipants (n = 319)	Standardized difference
Demographics						
Age group, y						
<18	16 (5.8)	26 (9.3)	−0.134^a^	1 (0.3)	9 (2.8)	−0.202^a^
18-64	232 (84.1)	193 (70.0)	0.339^b^	118 (37.0)	112 (35.2)	0.036
≥65	28 (10.1)	57 (20.7)	−0.294^a^	200 (62.7)	198 (61.9)	0.016
Sex						
Male	165 (59.8)	153 (55.4)	0.089	217 (68.0)	173 (54.2)	0.282^b^
Female	111 (40.2)	123 (44.6)	−0.089	102 (32.0)	146 (45.8)	−0.282^a^
Ethnicity						
Hispanic or Latino	40 (14.5)	49 (17.9)	−0.093	51 (16.0)	42 (13.1)	0.081
Not Hispanic or Latino	126 (45.7)	114 (41.2)	0.090	118 (37.0)	140 (43.8)	−0.139^a^
Unknown	110 (39.9)	113 (40.9)	−0.021	150 (47.0)	137 (43.1)	0.079
Race						
White	134 (48.6)	104 (37.7)	0.221^b^	141 (44.2)	134 (41.9)	0.046
Black or African American	15 (5.4)	31 (11.4)	−0.216^a^	32 (10.0)	33 (10.3)	−0.007
Other^c^	7 (2.5)	8 (2.8)	−0.014	15 (4.7)	8 (2.6)	0.114^b^
Unknown	120 (43.5)	133 (48.2)	−0.094	131 (41.1)	144 (45.2)	−0.084
Comorbidities						
Acute respiratory disease	22 (8.0)	36 (13.0)	−0.163^a^	20 (6.3)	46 (14.4)	−0.269^a^
Chronic liver disease	147 (53.3)	146 (52.7)	0.012	4 (1.3)	13 (4.1)	−0.173^a^
Chronic obstructive lung disease	10 (3.6)	22 (8.1)	−0.191^a^	27 (8.5)	34 (10.8)	−0.077
Depressive disorder	29 (10.5)	44 (15.9)	−0.161^a^	26 (8.2)	31 (9.7)	−0.052
Diabetes mellitus	32 (11.6)	57 (20.8)	−0.252^a^	106 (33.2)	106 (33.3)	−0.002
Gastroesophageal reflux disease	13 (4.7)	31 (11.2)	−0.241^a^	36 (11.3)	39 (12.1)	−0.026
Hyperlipidemia	41 (14.9)	57 (20.6)	−0.150^a^	165 (51.7)	164 (51.6)	0.003
Hypertensive disorder	82 (29.7)	114 (41.4)	−0.246^a^	200 (62.7)	220 (68.9)	−0.132^a^
Lesion of liver	88 (31.9)	88 (31.8)	0.001	1 (0.3)	8 (2.5)	−0.189^a^
Obesity	11 (4.0)	26 (9.6)	−0.223^a^	36 (11.3)	33 (10.5)	0.026
Osteoarthritis	24 (8.7)	35 (12.6)	−0.127^a^	36 (11.3)	47 (14.7)	−0.102^a^
Pneumonia	21 (7.6)	33 (12.1)	−0.150^a^	24 (7.5)	43 (13.3)	−0.192^a^
Renal impairment	63 (22.8)	84 (30.4)	−0.172^a^	58 (18.2)	87 (27.4)	−0.220^a^
Urinary tract infectious diseases	10 (3.6)	22 (7.8)	−0.183^a^	11 (3.4)	24 (7.6)	−0.186^a^
Viral hepatitis C infection	147 (53.3)	141 (51.1)	0.043	3 (0.9)	0	0.135^b^
Visual system disorder	17 (6.2)	30 (10.8)	−0.167^a^	34 (10.7)	40 (12.5)	−0.056
Atrial fibrillation	9 (3.3)	17 (6.0)	−0.130^a^	97 (30.4)	90 (28.2)	0.048
Cerebrovascular disease	9 (3.3)	16 (5.7)	−0.115^a^	44 (13.8)	80 (25.1)	−0.289^a^
Coronary arteriosclerosis	14 (5.1)	20 (7.2)	−0.088	121 (37.9)	121 (37.9)	0.001
Heart disease	62 (22.5)	84 (30.3)	−0.177^a^	284 (89.0)	276 (86.5)	0.077
Heart failure	9 (3.3)	27 (9.9)	−0.268^a^	164 (51.4)	153 (47.8)	0.072
Ischemic heart disease	5 (1.8)	18 (6.6)	−0.241^a^	131 (41.1)	94 (29.4)	0.247^b^
Peripheral vascular disease	2 (0.7)	0	0.119^b^	92 (28.8)	91 (28.6)	0.003
Venous thrombosis	9 (3.3)	13 (4.9)	−0.080	10 (3.1)	16 (5.0)	−0.096
Hematologic neoplasm	0	0	0	3 (0.9)	7 (2.3)	−0.111^a^
Malignant lymphoma	2 (0.7)	0	0.119^b^	5 (1.6)	5 (1.6)	−0.001
Malignant neoplastic disease	48 (17.4)	45 (16.2)	0.033	21 (6.6)	41 (13.0)	−0.217^a^
Malignant tumor of breast	2 (0.7)	0	0.119^b^	3 (0.9)	7 (2.2)	−0.106^a^
Malignant tumor of lung	1 (0.4)	0	0.090	0	0	0.000
Malignant tumor of urinary bladder	0	0	0	1 (0.3)	0	0.078
Primary malignant neoplasm of prostate	1 (0.4)	0	0.090	3 (0.9)	0	0.135^b^
Medication use						
Agents acting on the renin-angiotensin system	49 (17.8)	74 (26.7)	−0.215^a^	196 (61.4)	179 (56.1)	0.109^b^
Antibacterial medications for systemic use	140 (50.7)	165 (59.7)	−0.182^a^	149 (46.7)	172 (53.8)	−0.143^a^
Antidepressant medications	44 (15.9)	64 (23.1)	−0.183^a^	48 (15.0)	67 (21.1)	−0.160^a^
Antiepileptic medications	17 (6.2)	42 (15.4)	−0.299^a^	50 (15.7)	74 (23.3)	−0.194^a^
Anti-inflammatory and antirheumatic products	77 (27.9)	96 (34.6)	−0.146^a^	67 (21.0)	65 (20.3)	0.018
Antineoplastic agents	24 (8.7)	27 (9.8)	−0.037	5 (1.6)	14 (4.5)	−0.167^a^
Antithrombotic agents	99 (35.9)	127 (46.0)	−0.207^a^	266 (83.4)	238 (74.5)	0.220^b^
β blocking agents	63 (22.8)	86 (31.2)	−0.189^a^	207 (64.9)	203 (63.6)	0.028
Calcium channel blockers	46 (16.7)	61 (22.0)	−0.133^a^	110 (34.5)	114 (35.7)	−0.025
Diuretic medications	77 (27.9)	92 (33.4)	−0.120^a^	202 (63.3)	190 (59.4)	0.080
Drugs for acid-related disorders	102 (37.0)	139 (50.4)	−0.272^a^	153 (48.0)	186 (58.2)	−0.206^a^
Drugs for obstructive airway diseases	38 (13.8)	70 (25.2)	−0.290^a^	86 (27.0)	106 (33.3)	−0.138^a^
Drugs used in diabetes	47 (17.0)	68 (24.6)	−0.188^a^	119 (37.3)	117 (36.8)	0.010
Immunosuppressant medications	74 (26.8)	63 (22.8)	0.092	10 (3.1)	26 (8.2)	−0.223^a^
Lipid-modifying agents	33 (12.0)	57 (20.7)	−0.236^a^	210 (65.8)	188 (59.1)	0.139^b^
Opioids	108 (39.1)	126 (45.6)	−0.131^a^	98 (30.7)	118 (37.0)	−0.134^a^
Psycholeptic medications	69 (25.0)	98 (35.6)	−0.233^a^	93 (29.2)	118 (37.1)	−0.169^a^

^a^ Covariate was more prevalent among nonparticipants than participants (ie, standardized difference ≤−0.1).

^b^ Covariate was more prevalent among participants than nonparticipants (ie, standardized difference ≥0.1).

^c^ This category consists of any reported race that did not qualify for 1 of the other categories (ie, the reported race was not White, Black or African American, or Unknown).

For comorbidities, participants generally had fewer underlying conditions across all trials. Among the 31 conditions, participants had substantially lower prevalence of conditions compared with their nonparticipant counterparts in neoplastic trials (64.5% [20 conditions]), with the largest difference being for hypertensive disorder (234 [31.8%] vs 315 [42.7%]); in digestive system trials (61.3% [19]), with the largest difference being for hyperlipidemia (33 [10.3%] vs 65 [20.3%]); in inflammatory disorder trials (58.1% [18]), with the largest difference being for heart failure (9 [3.3%] vs 27 [9.9%]); and in CV system trials (38.7% [12]), with the largest difference being for cerebrovascular disease (44 [13.8%] vs 80 [25.1%]). In contrast, nonparticipants had substantially lower prevalence of underlying conditions in neoplastic trials (6.4% [2 conditions]), in digestive system trials (6.4% [2]), in inflammatory disorder trials (9.7% [3]), and in CV system trials (9.7% [3]). In neoplastic trials in particular, after hypertension, the largest differences between trial participants and nonparticipants were found for prevalence of heart disease (26.6% vs 36.9%), renal impairment (9.8% vs 17.3%), ischemic heart disease (1.8% vs 5.5%), and coronary arteriosclerosis (6.8% vs 12.4%), indicating that the largest differences tend to be for CV diseases. Consequently, for CV trials, there was a lower prevalence of malignant neoplastic disease between trial participants and nonparticipants (6.6% vs 13.0%).

For medication history, trial participants generally had fewer prescriptions than nonparticipants within the 17 medication classes assessed. Participants had substantially lower prevalence of prescriptions compared with nonparticipants in neoplastic trials (64.7% [11 medication classes]), with the largest difference being for antithrombotic agents (305 [41.4%] vs 397 [53.9%]); in digestive system trials (58.8% [10]), with the largest difference being for drugs for treatment of obstructive airway diseases (39 [12.1%] vs 75 [23.5%]); in inflammatory disorder trials (88.2% [15]), with the largest difference being for antiepileptics (17 [6.2%] vs 42 [15.4%]); and in CV system trials (52.9% [9]), with the largest difference being for immunosuppressants (10 [3.1%] vs 26 [8.2%]). In contrast, nonparticipants had substantially lower prescriptions than participants in digestive trials (5.9% [1 medication class]) and in CV system trials (17.6% [3]).

### Association of Trial Participant Covariates and Trial Characteristics

**Figure 1.**
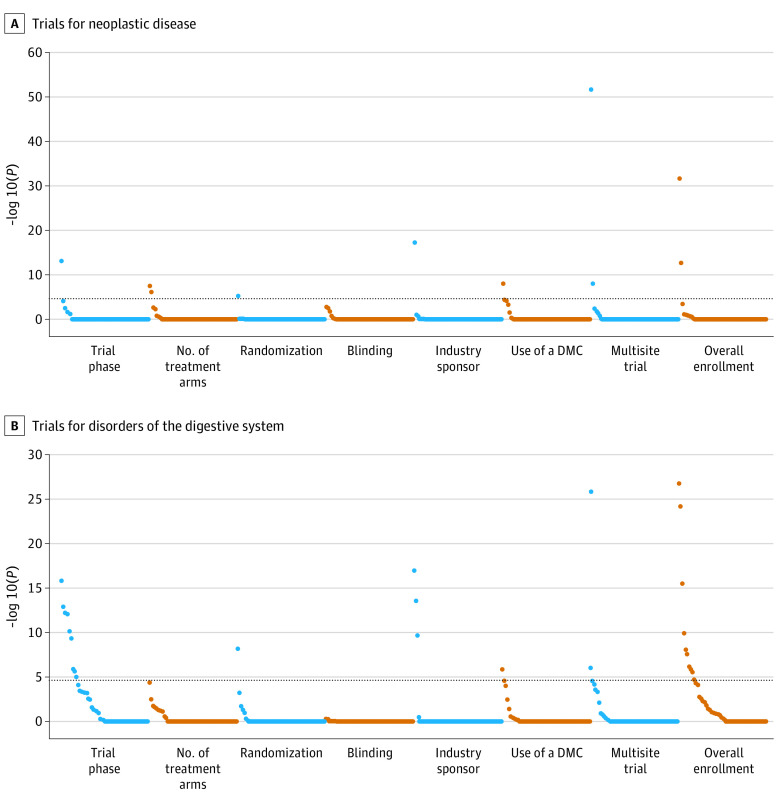
Associations Between Trial Participant Covariates and Trial Characteristics in Trials for Neoplastic Disease and Disorders of the Digestive System Covariates above the dashed line are statistically significant. Covariates with the most statistically significant associations per each trial characteristic are as follows. A, Participant age with trial phase, number of treatment arms, and industry sponsorship; malignant tumor of urinary bladder with multisite trials and overall enrollment; malignant tumor of lung with use of a data monitoring committee (DMC); and use of opioids with randomization. B, Malignant neoplastic disease with trial phase and overall enrollment; antithrombotic agents with industry sponsorship; primary malignant neoplasm of prostate with randomization; immunosuppressant medications with use of a DMC; and heart disease with multisite trial.

**Figure 2.**
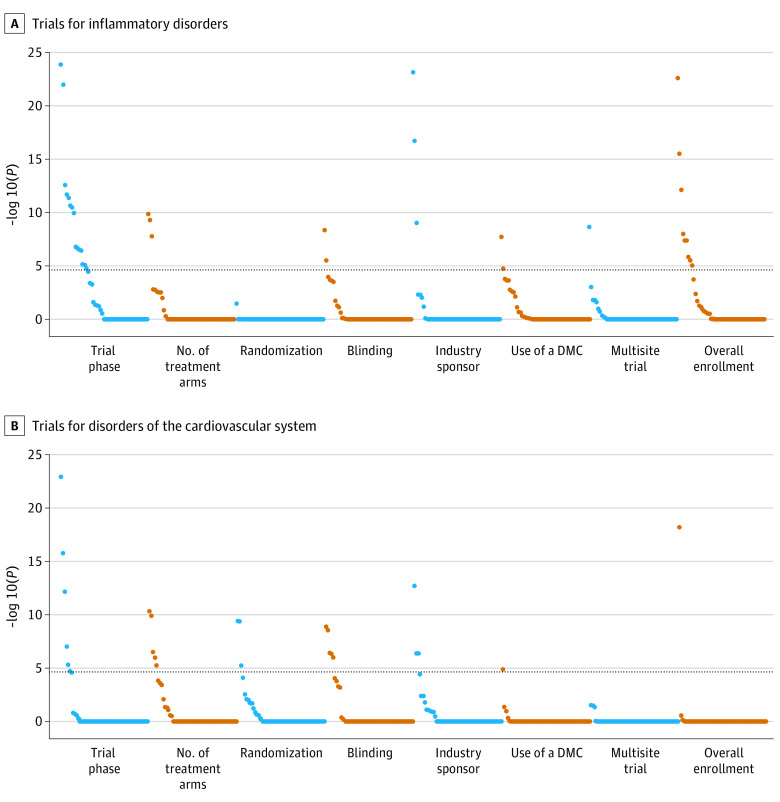
Associations Between Trial Participant Covariates and Trial Characteristics in Trials for Inflammatory Disorders and Disorders of the Cardiovascular System Covariates above the dashed line are statistically significant. Covariates with the most statistically significant associations per each trial characteristic are as follows. A, Viral hepatitis C infection with trial phase; heart disease with blinding; renal impairment with multisite trials and overall enrollment; antithrombotic agents with number of treatment arms and industry sponsorship; and immunosuppressant medications with use of a data monitoring committee (DMC). B, Age with randomization; peripheral vascular disease with trial phase and overall enrollment; atrial fibrillation with number of treatment arms; hyperlipidemia with blinding; heart disease with industry sponsorship; and heart failure with use of a DMC.

Figure 1 and Figure 2 show the associations between trial participants’ covariates and trial characteristics for each disease domain (data for each individual data point are shown in eTables 3-6 in the Supplement). Neoplastic disease trials had the fewest statistically significant associations; the most prominent associations were for (1) malignant tumor of urinary bladder and multisite trials and overall enrollment and (2) age and phase, number of treatment arms, and industry sponsorship. Regarding age associations specifically, for industry sponsorship, there was a negative association between the inclusion of children younger than 18 years in a trial and industry-sponsor funding (odds ratio, 0.14; 95% CI, 0.09-0.25); 33 of the 69 children (47.8%) included in this study were part of an industry-sponsored trial, compared with 575 of 668 adults (86.1%). For trial phase, no children were involved in phase 1 trials; this is in contrast to 90 of 331 adults (27.2%) aged 18 to 64 years and 63 of 337 elderly participants (18.7%) aged 65 years or older who participated in a phase 1 trial. Among the 26 statistically significant associations for digestive system trials and the 36 statistically significant associations for inflammatory disorder trials, 18 associations overlapped between the 2 disease domains. For CV system trials, the most statistically significant associations were for peripheral vascular disease and phase and overall enrollment.

## Discussion

In this cross-sectional study, we used a novel combination of EHR data and trial enrollment data to compare trial participants and nonparticipants, and we examined associations between participant covariates and trial parameters. We found that trial participants had fewer comorbidities and fewer medication prescriptions than did nonparticipants across 4 different disease domains, similar to the findings of prior work.^2,3,4,5,6^ We also found statistically significant associations among a variety of participant covariates and trial parameters.

In neoplastic disease trials, trial participants had fewer comorbidities than nonparticipants. The largest differences between trial participants and nonparticipants were found for hypertensive disorder (31.8% of participants vs 42.7% of nonparticipants), heart disease (26.6% vs 36.9%), renal impairment (9.8% vs 17.3%), ischemic heart disease (1.8% vs 5.5%), and coronary arteriosclerosis (6.8% vs 12.4%). The observations for CV disease may be associated with CV-related exclusion criteria^20^ because many cancer therapies are associated with CV toxic effects.^21^ However, given the large prevalence of trial nonparticipants who had a CV comorbidity, this finding suggests a need for trials expressly focused on this subpopulation to find safer therapeutic alternatives; none of the neoplastic disease trials included in this study qualified as a CV system trial.

Regarding associations between participant covariates and trial parameters, 2 prominent findings were an association between participant age and industry sponsorship and between participant age and trial phase. Industry sponsors might be cautious about funding pediatric trials because of increased liability, more restrictive regulatory oversight, and minimal financial gain.^22,23,24^ Regarding trial phase, the most prominent observation was in phase 1 trials, in which no children were involved. Phase 1 trials typically focus on initial safety assessments of interventions given to humans for the first time, usually to establish a maximum tolerated dose.^25,26,27^ Despite no such pediatric trials in this study’s data, some trials were designated as phase 1/phase 2, in which finding the maximum tolerated dose was incorporated as part of the study design for ultimately assessing efficacy. This hybrid design may be particularly important for the pediatric population because it allows for a timelier evaluation of efficacy while also attempting to mitigate potential toxic effects.^28^

Digestive system disorders and inflammatory disorders were the second and third most common disease domains for which trials were conducted, but the prevalence of trials in both domains was primarily focused on hepatitis C virus; approximately half of the trial participants in both disease domains had viral hepatitis C infection. Subsequently, the 2 sets of covariate differences observed among trial participants and nonparticipants in these disease domains were fairly similar albeit with differing magnitudes for each covariate. One possible explanation is that many of the hepatitis C trials included in this study required a surgical component (ie, liver transplant). Individuals undergoing such a procedure are required to display adequate health, such as having no severe CV disease or no severe renal dysfunction, to ensure tolerance of postsurgery medications and to minimize concerns that may jeopardize the success of the procedure.^29^ This is supported by the finding in this study of a higher prevalence of immunosuppressant medication use in the trial participant groups (although there was a discrepancy between the number of immunosuppressant medications and the diagnoses of viral hepatitis C infection, this may reflect pretransplant vs posttransplant timing of trial initiation). Many of the associations observed in these 2 disease domains were found in the hepatitis C trials. For example, many of the viral hepatitis C trials were phase 2 trials, and thus many covariates in these trials were significantly associated with trial phase, including viral hepatitis C, chronic liver disease, lesions of the liver, and use of immunosuppressant medications.

Although CV system trials had the fewest prominent differences between participants and nonparticipants, observations consistent with prior studies persisted,^3,6^ particularly for differences in the prevalence of cerebrovascular disease (present in 13.8% of trial participants vs 25.1% of nonparticipants) and malignant neoplastic disease (present in 6.6% of trial participants vs 13.0% of nonparticipants) as well as in female participation (32.0% of trial participants vs 45.8% of nonparticipants). The difference in the prevalence of cerebrovascular disease might be a result of individuals experiencing a cerebrovascular event that led to cognitive impairment or a debilitating disability, thus precluding trial participation.^30,31,32^ Alternatively, the difference might result from some trials designating this covariate as a safety outcome, which would exclude individuals with cerebrovascular disease because they would begin the trial at increased risk.^33,34^ Regardless, one-fourth of nonparticipants were found to have prior cerebrovascular disease, and overlooking these individuals in trials may hinder how relevant the results are to this group. Regarding the low prevalence of female participation, a possible explanation is that females may perceive greater risk in trial participation than males do, and thus, they may forgo participation; another possibility is that females present with CV disease at later ages, which may exclude them from certain trials.^3,35,36,37,38^ In addition, the difference in the prevalence of malignant neoplastic disease among participants and nonparticipants in CV system trials echoes the aforementioned concern that some chemotherapy regimens may cause cardiotoxic effects.^21^

### Limitations

This study has limitations. First, there was potential misclassification when selecting nonparticipants. In particular, matching on conditions did not consider trials defined by multiple simultaneous conditions. Likewise, EHR data are susceptible to data-quality concerns, such as missing data elements and erroneous documentation of irrelevant elements, that can affect how patients are assessed.^39^ We tried to mitigate these concerns by matching trial participants and nonparticipants based on condition, calendar time, and number of visits to a health care professional. Second, trial characteristics were based on ClinicalTrials.gov entries, which represent a condensed summary of protocols.^40^ Third, we had access to patients from only a single center, resulting in having information regarding only some of the participants in multisite trials. Fourth, the study data were from a single large academic medical center, which may have affected the types of patients available and the types of trials conducted. For example, the CUIMC houses many specialty services, such as oncology clinics and transplant centers, for patients with complex medical histories, which may have led to data from a higher proportion of patients who had greater comorbidity burdens compared with patients in other clinical environments.

## Conclusions

In this cross-sectional study, combining data on prior trial enrollment with EHR data provided a source of information to evaluate the generalizability of trials and to inform the designs of future trials. The findings of this analysis support prior observations, highlight potentially overlooked subgroups, and provide insight regarding why certain patient characteristics may be associated with certain trial characteristics. The results also suggest that linking EHR data with data on prior trial enrollment may enhance the interpretation of clinical trial findings.

## Notes

Supplement. **eFigure.** Overview of primary methodology **eTable 1.** Attrition of trials and participants **eTable 2.** Covariate comparisons between trial participants and nonparticipants (all trials) **eTable 3.** Associations between trial participants’ covariates and trial characteristics, neoplastic disease trials **eTable 4.** Associations between trial participants’ covariates and trial characteristics, disorder of digestive system trials **eTable 5.** Associations between trial participants’ covariates and trial characteristics, inflammatory disorder trials **eTable 6.** Associations between trial participants’ covariates and trial characteristics, disorder of cardiovascular system trials
