# Supplementary material for: Comparison of Clinical Characteristics Between Clinical Trial Participants and Nonparticipants Using Electronic Health Record Data
Source: JAMA Netw Open. 2021 Apr 7;4(4):e214732. doi: 10.1001/jamanetworkopen.2021.4732 (PMC8027910; doi:10.1001/jamanetworkopen.2021.4732)
Supplement: Supplement. — eFigure. Overview of primary methodology eTable 1. Attrition of trials and participants eTable 2. Covariate comparisons between trial participants and nonparticipants (all trials) eTable 3. Associations between trial participants’ covariates and trial characteristics, neoplastic disease trials eTable 4. Associations between trial participants’ covariates and trial characteristics, disorder of digestive system trials eTable 5. Associations between trial participants’ covariates and trial characteristics, inflammatory disorder trials eTable 6. Associations between trial participants’ covariates and trial characteristics, disorder of cardiovascular system trials [file jamanetwopen-e214732-s001.pdf]

Supplementary Online Content

Rogers JR, Liu C, Hripcsak G, Cheung YK, Weng C. Comparison of clinical characteristics between clinical trial participants and nonparticipants using electronic health record data. *JAMA Netw Open*. 2021;4(4):e214732. doi:10.1001/jamanetworkopen.2021.4732.

eFigure. Overview of primary methodology

eTable 1. Attrition of trials and participants

eTable 2. Covariate comparisons between trial participants and nonparticipants (all trials)

eTable 3. Associations between trial participants' covariates and trial characteristics, neoplastic disease trials

eTable 4. Associations between trial participants' covariates and trial characteristics, disorder of digestive system trials

eTable 5. Associations between trial participants' covariates and trial characteristics, inflammatory disorder trials

eTable 6. Associations between trial participants' covariates and trial characteristics, disorder of cardiovascular system trials

This supplementary material has been provided by the authors to give readers additional information about their work.

© 2021 Rogers JR et al. *JAMA Network Open*.

eFigure: Overview of primary methodology[a]

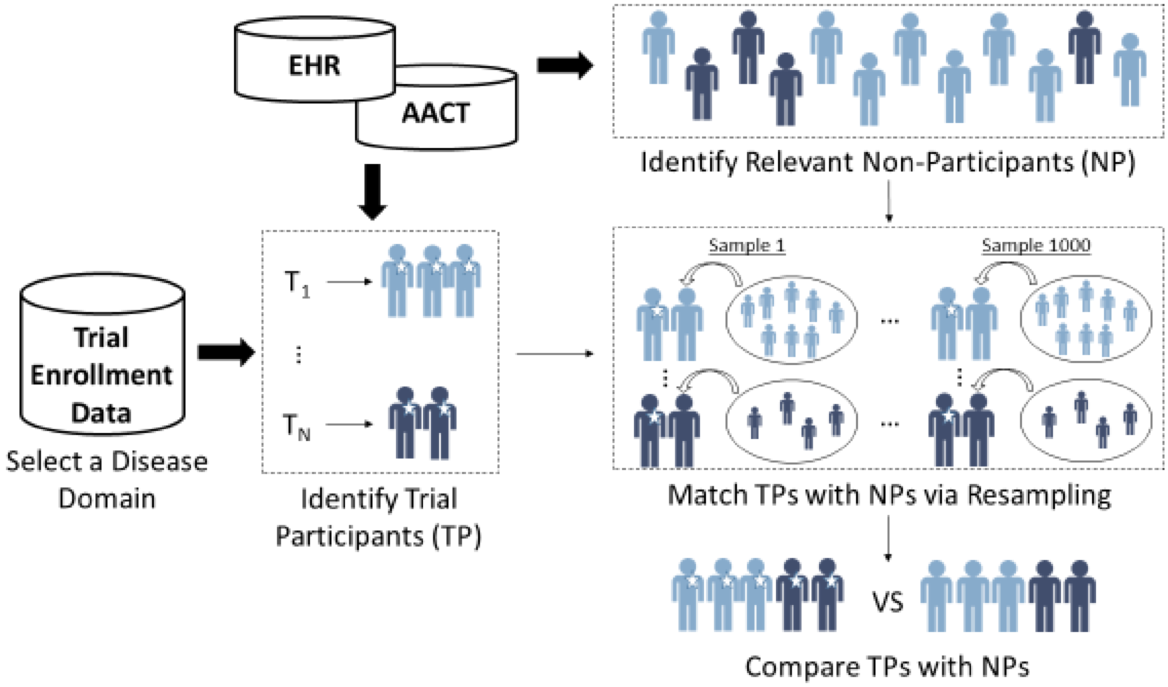

[a]EHR = electronic health record data; AACT = Aggregate Analysis of ClinicalTrials.gov data; individuals with a star indicate trial participants. Matching between a trial participant and a non-participant is based on the same index condition, calendar month and year, and number of prior visit occurrences. To compare non-participants to participants, the estimates of the non-participants were aggregated by taking the mean of the estimates for each characteristic

© 2021 Rogers JR et al. *JAMA Network Open*.

eTable 1: Attrition of trials and participants

Criteria	Trials Excluded[a]	Remaining Number of Trials	Participants Excluded	Remaining Number of Participants
Starting Point		297		4022
Condition description(s) of trials did not have standard code to map to	39	258	898	3124
Participants did not have a relevant standard code within 365 days of their trial status date	35	223	764	2360
Participants did not have a relevant standard code within 365 days of their newly assigned index date	9	214	370	1990
Participants could not be matched to at least one non-participant during each resampling	12	202	345	1645

[a]For criteria specific to participant removal, a trial is excluded if all available participants are excluded as a result of the applied criterion

© 2021 Rogers JR et al. *JAMA Network Open*.

eTable 2: Covariate comparisons between trial participants and nonparticipants (all trials)[a]

	Overall (N = 1645 each)		
	P	NP	StDiff
Demographics			
Age group			
Younger than 18 years	138 (8.4)	140 (8.5)	-0.003
18 to 64 years	854 (51.9)	785 (47.7)	0.084
65 years and older	653 (39.7)	720 (43.8)	-0.083
Sex			
Male	929 (56.5)	855 (52.0)	0.091
Female	716 (43.5)	790 (48.0)	-0.091
Ethnicity			
Hispanic or Latino	214 (13.0)	241 (14.7)	-0.048
Not Hispanic or Latino	744 (45.2)	751 (45.6)	-0.008
Unknown	687 (41.8)	653 (39.7)	0.043
Race			
White	776 (47.2)	710 (43.2)	0.080
Black or African American	126 (7.7)	155 (9.4)	-0.063
Other[d]	61 (3.7)	58 (3.5)	0.009
Unknown	682 (41.5)	721 (43.8)	-0.048
Comorbidities			
Acute respiratory disease	127 (7.7)	214 (13.0)	-0.174[b]
Chronic liver disease	192 (11.7)	220 (13.4)	-0.050
Chronic obstructive lung disease	82 (5.0)	137 (8.3)	-0.134[b]
Depressive disorder	127 (7.7)	189 (11.5)	-0.128[b]
Diabetes mellitus	313 (19.0)	399 (24.3)	-0.128[b]
Gastroesophageal reflux disease	153 (9.3)	213 (12.9)	-0.116[b]
Hyperlipidemia	426 (25.9)	498 (30.3)	-0.097
Hypertensive disorder	609 (37.0)	771 (46.8)	-0.201[b]
Lesion of liver	242 (14.7)	195 (11.8)	0.085
Obesity	97 (5.9)	126 (7.7)	-0.071
Osteoarthritis	176 (10.7)	249 (15.1)	-0.132[b]
Pneumonia	143 (8.7)	233 (14.2)	-0.172[b]
Renal impairment	243 (14.8)	364 (22.2)	-0.190[b]
Urinary tract infectious disease	87 (5.3)	164 (10.0)	-0.176[b]
Viral hepatitis C	165 (10.0)	171 (10.4)	-0.014
Visual system disorder	173 (10.5)	220 (13.3)	-0.088
Atrial fibrillation	155 (9.4)	194 (11.8)	-0.079
Cerebrovascular disease	110 (6.7)	172 (10.4)	-0.134[b]
Coronary arteriosclerosis	219 (13.3)	265 (16.1)	-0.079
Heart disease	637 (38.7)	748 (45.5)	-0.137[b]

© 2021 Rogers JR et al. *JAMA Network Open*.

	Overall (N = 1645 each)		
Heart failure	239 (14.5)	288 (17.5)	-0.081
Ischemic heart disease	176 (10.7)	185 (11.2)	-0.017
Peripheral vascular disease	110 (6.7)	129 (7.8)	-0.044
Venous thrombosis	63 (3.8)	102 (6.2)	-0.111[b]
Hematologic neoplasm	329 (20.0)	338 (20.5)	-0.013
Malignant lymphoma	179 (10.9)	187 (11.4)	-0.015
Malignant neoplastic disease	836 (50.8)	872 (53.0)	-0.044
Malignant tumor of breast	105 (6.4)	132 (8.0)	-0.063
Malignant tumor of lung	102 (6.2)	73 (4.4)	0.078
Malignant tumor of urinary bladder	64 (3.9)	69 (4.2)	-0.014
Primary malignant neoplasm of prostate	77 (4.7)	73 (4.5)	0.011
Medication use			
Agents acting on the renin-angiotensin system	490 (29.8)	540 (32.8)	-0.065
Antibacterials for systemic use	849 (51.6)	975 (59.2)	-0.154[b]
Antidepressants	290 (17.6)	341 (20.7)	-0.079
Antiepileptics	281 (17.1)	354 (21.5)	-0.112[b]
Antiinflammatory and antirheumatic products	406 (24.7)	457 (27.8)	-0.071
Antineoplastic agents	329 (20.0)	410 (24.9)	-0.118[b]
Antithrombotic agents	785 (47.7)	898 (54.6)	-0.139[b]
Beta blocking agents	495 (30.1)	584 (35.5)	-0.116[b]
Calcium channel blockers	341 (20.7)	411 (25.0)	-0.103[b]
Diuretics	510 (31.0)	589 (35.8)	-0.102[b]
Drugs for acid related disorders	734 (44.6)	922 (56.0)	-0.230[b]
Drugs for obstructive airway diseases	344 (20.9)	465 (28.3)	-0.172[b]
Drugs used in diabetes	368 (22.4)	445 (27.0)	-0.108[b]
Immunosuppressants	174 (10.6)	222 (13.5)	-0.089
Lipid modifying agents	510 (31.0)	568 (34.5)	-0.076
Opioids	678 (41.2)	767 (46.6)	-0.110[b]
Psycholeptics	586 (35.6)	682 (41.4)	-0.120[b]

[a]P = participants; NP = non-participants; StDiff = standardized differences
[b]Covariate is more prevalent in NP than P (i.e. standardized difference < -0.1)
[c]Covariate is more prevalent in P than NP (i.e. standardized difference > 0.1), of which none were found for this particular table
[d]This category consists of any reported race that doesn't qualify for one of the other categories (i.e. record of race was not "White", "Black or African American", or "Unknown")

© 2021 Rogers JR et al. *JAMA Network Open*.

eTable 3: Associations between trial participants' covariates and trial characteristics, neoplastic disease trials

Trial Characteristic	Covariate Label	Statistical Test	P-value	Adjusted P-value
Phase	Age	Fisher	2.19E-16	8.92E-14
Phase	Malignant Tumor Of Breast	Fisher	2.45E-07	1E-04
Phase	Malignant Tumor Of Urinary Bladder	Fisher	8.6E-06	0.003507
Phase	Hematologic Neoplasm	Fisher	6.53E-05	0.026641
Phase	Lesion Of Liver	Fisher	7.35E-05	0.029971
Phase	Viral Hepatitis C	Fisher	0.00018	0.073527
Phase	Female	Fisher	0.875367	1
Phase	Osteoarthritis	Fisher	0.008318	1
Phase	Urinary Tract Infectious Disease	Fisher	0.013091	1
Phase	Primary Malignant Neoplasm Of Prostate	Fisher	0.003044	1
Phase	Diabetes Mellitus	Fisher	0.788566	1
Phase	Chronic Obstructive Lung Disease	Fisher	0.315865	1
Phase	Pneumonia	Fisher	0.736598	1
Phase	Atrial Fibrillation	Fisher	0.099467	1
Phase	Heart Failure	Fisher	0.724192	1
Phase	Hypertensive Disorder	Fisher	0.049674	1
Phase	Coronary Arteriosclerosis	Fisher	0.984993	1
Phase	Gastroesophageal Reflux Disease	Fisher	0.036609	1
Phase	Peripheral Vascular Disease	Fisher	0.237273	1
Phase	Heart Disease	Fisher	0.991886	1
Phase	Cerebrovascular Disease	Fisher	0.153876	1
Phase	Malignant Lymphoma	Fisher	0.996852	1
Phase	Hyperlipidemia	Fisher	0.827774	1
Phase	Obesity	Fisher	0.302778	1
Phase	Depressive Disorder	Fisher	0.648586	1
Phase	Malignant Tumor Of Lung	Fisher	0.01462	1
Phase	Venous Thrombosis	Fisher	0.121116	1
Phase	Acute Respiratory Disease	Fisher	0.30076	1
Phase	Renal Impairment	Fisher	0.299393	1
Phase	Visual System Disorder	Fisher	0.224866	1
Phase	Ischemic Heart Disease	Fisher	0.664786	1
Phase	Chronic Liver Disease	Fisher	0.014001	1
Phase	Drugs For Acid Related Disorders	Fisher	0.717589	1
Phase	Drugs Used In Diabetes	Fisher	0.67405	1
Phase	Antithrombotic Agents	Fisher	0.33854	1
Phase	Antineoplastic Agents	Fisher	0.033431	1
Phase	Diuretics	Fisher	0.837868	1

© 2021 Rogers JR et al. *JAMA Network Open*.

Trial Characteristic	Covariate Label	Statistical Test	P-value	Adjusted P-value
Phase	Beta Blocking Agents	Fisher	0.799427	1
Phase	Calcium Channel Blockers	Fisher	0.036302	1
Phase	Agents Acting On The Renin-Angiotensin System	Fisher	0.072965	1
Phase	Lipid Modifying Agents	Fisher	0.915152	1
Phase	Antibacterials For Systemic Use	Fisher	0.16751	1
Phase	Drugs For Obstructive Airway Diseases	Fisher	0.951011	1
Phase	Immunosuppressants	Fisher	0.05703	1
Phase	Antiinflammatory And Antirheumatic Products	Fisher	0.016927	1
Phase	Opioids	Fisher	0.009059	1
Phase	Antiepileptics	Fisher	0.083989	1
Phase	Psycholeptics	Fisher	0.068029	1
Phase	Antidepressants	Fisher	0.003726	1
Phase	Ethnicity	Fisher	0.652108	1
Phase	Race	Fisher	0.47422	1
Number of Treatment Arms	Age	Chi-squared	8.12E-11	3.31E-08
Number of Treatment Arms	Malignant Tumor Of Urinary Bladder	Chi-squared	1.92E-09	7.82E-07
Number of Treatment Arms	Urinary Tract Infectious Disease	Chi-squared	5.77E-06	0.002352
Number of Treatment Arms	Opioids	Chi-squared	1.19E-05	0.004859
Number of Treatment Arms	Antineoplastic Agents	Chi-squared	0.000408	0.166593
Number of Treatment Arms	Drugs For Acid Related Disorders	Chi-squared	0.00075	0.305965
Number of Treatment Arms	Primary Malignant Neoplasm Of Prostate	Chi-squared	0.000931	0.379781
Number of Treatment Arms	Malignant Lymphoma	Chi-squared	0.002182	0.890429
Number of Treatment Arms	Female	Chi-squared	0.059422	1
Number of Treatment Arms	Osteoarthritis	Chi-squared	0.148542	1
Number of Treatment Arms	Viral Hepatitis C	Fisher	0.009384	1
Number of Treatment Arms	Diabetes Mellitus	Chi-squared	0.567191	1
Number of Treatment Arms	Chronic Obstructive Lung Disease	Chi-squared	0.971757	1
Number of Treatment Arms	Pneumonia	Chi-squared	0.759322	1
Number of Treatment Arms	Atrial Fibrillation	Chi-squared	0.775424	1

© 2021 Rogers JR et al. *JAMA Network Open*.

Trial Characteristic	Covariate Label	Statistical Test	P-value	Adjusted P-value
Number of Treatment Arms	Heart Failure	Chi-squared	0.036782	1
Number of Treatment Arms	Hypertensive Disorder	Chi-squared	0.67676	1
Number of Treatment Arms	Coronary Arteriosclerosis	Chi-squared	0.743851	1
Number of Treatment Arms	Gastroesophageal Reflux Disease	Chi-squared	0.863159	1
Number of Treatment Arms	Peripheral Vascular Disease	Fisher	0.665762	1
Number of Treatment Arms	Heart Disease	Chi-squared	0.003259	1
Number of Treatment Arms	Cerebrovascular Disease	Chi-squared	0.830617	1
Number of Treatment Arms	Hyperlipidemia	Chi-squared	0.706501	1
Number of Treatment Arms	Obesity	Chi-squared	0.769683	1
Number of Treatment Arms	Depressive Disorder	Chi-squared	0.271038	1
Number of Treatment Arms	Malignant Tumor Of Lung	Chi-squared	0.01166	1
Number of Treatment Arms	Venous Thrombosis	Chi-squared	0.999186	1
Number of Treatment Arms	Acute Respiratory Disease	Chi-squared	0.745193	1
Number of Treatment Arms	Renal Impairment	Chi-squared	0.911955	1
Number of Treatment Arms	Hematologic Neoplasm	Chi-squared	0.532793	1
Number of Treatment Arms	Lesion Of Liver	Chi-squared	0.586726	1
Number of Treatment Arms	Malignant Tumor Of Breast	Chi-squared	0.049856	1
Number of Treatment Arms	Visual System Disorder	Chi-squared	0.332422	1
Number of Treatment Arms	Ischemic Heart Disease	Fisher	0.289349	1
Number of Treatment Arms	Chronic Liver Disease	Chi-squared	0.391975	1
Number of Treatment Arms	Drugs Used In Diabetes	Chi-squared	0.576443	1
Number of Treatment Arms	Antithrombotic Agents	Chi-squared	0.088931	1
Number of Treatment Arms	Diuretics	Chi-squared	0.051144	1
Number of Treatment Arms	Beta Blocking Agents	Chi-squared	0.364282	1

© 2021 Rogers JR et al. *JAMA Network Open*.

Trial Characteristic	Covariate Label	Statistical Test	P-value	Adjusted P-value
Number of Treatment Arms	Calcium Channel Blockers	Chi-squared	0.002992	1
Number of Treatment Arms	Agents Acting On The Renin-Angiotensin System	Chi-squared	0.115568	1
Number of Treatment Arms	Lipid Modifying Agents	Chi-squared	0.630845	1
Number of Treatment Arms	Antibacterials For Systemic Use	Chi-squared	0.009217	1
Number of Treatment Arms	Drugs For Obstructive Airway Diseases	Chi-squared	0.528727	1
Number of Treatment Arms	Immunosuppressants	Chi-squared	0.058443	1
Number of Treatment Arms	Antiinflammatory And Antirheumatic Products	Chi-squared	0.717943	1
Number of Treatment Arms	Antiepileptics	Chi-squared	0.039804	1
Number of Treatment Arms	Psycholeptics	Chi-squared	0.142005	1
Number of Treatment Arms	Antidepressants	Chi-squared	0.172212	1
Number of Treatment Arms	Ethnicity	Chi-squared	0.014679	1
Number of Treatment Arms	Race	Chi-squared	0.011471	1
Randomization	Opioids	Chi-squared	1.56E-08	6.36E-06
Randomization	Antineoplastic Agents	Chi-squared	0.001571	0.641154
Randomization	Drugs For Acid Related Disorders	Chi-squared	0.001701	0.694007
Randomization	Primary Malignant Neoplasm Of Prostate	Chi-squared	0.002054	0.838132
Randomization	Antithrombotic Agents	Chi-squared	0.002307	0.941374
Randomization	Female	Chi-squared	0.020886	1
Randomization	Osteoarthritis	Chi-squared	0.037135	1
Randomization	Urinary Tract Infectious Disease	Chi-squared	0.05026	1
Randomization	Viral Hepatitis C	Fisher	0.009916	1
Randomization	Malignant Tumor Of Urinary Bladder	Chi-squared	0.185099	1
Randomization	Diabetes Mellitus	Chi-squared	0.103392	1
Randomization	Chronic Obstructive Lung Disease	Chi-squared	0.294739	1
Randomization	Pneumonia	Chi-squared	1	1
Randomization	Atrial Fibrillation	Chi-squared	1	1
Randomization	Heart Failure	Chi-squared	0.223339	1
Randomization	Hypertensive Disorder	Chi-squared	0.547922	1
Randomization	Coronary Arteriosclerosis	Chi-squared	0.499086	1
Randomization	Gastroesophageal Reflux Disease	Chi-squared	0.734756	1
Randomization	Peripheral Vascular Disease	Fisher	1	1
Randomization	Heart Disease	Chi-squared	0.849455	1

© 2021 Rogers JR et al. *JAMA Network Open*.

Trial Characteristic	Covariate Label	Statistical Test	P-value	Adjusted P-value
Randomization	Cerebrovascular Disease	Chi-squared	1	1
Randomization	Malignant Lymphoma	Chi-squared	0.00492	1
Randomization	Hyperlipidemia	Chi-squared	0.097853	1
Randomization	Obesity	Chi-squared	0.54842	1
Randomization	Depressive Disorder	Chi-squared	0.564596	1
Randomization	Malignant Tumor Of Lung	Chi-squared	0.984539	1
Randomization	Venous Thrombosis	Chi-squared	0.688995	1
Randomization	Acute Respiratory Disease	Chi-squared	0.808081	1
Randomization	Renal Impairment	Chi-squared	0.999579	1
Randomization	Hematologic Neoplasm	Chi-squared	0.288938	1
Randomization	Lesion Of Liver	Chi-squared	0.158988	1
Randomization	Malignant Tumor Of Breast	Chi-squared	0.016233	1
Randomization	Visual System Disorder	Chi-squared	0.398611	1
Randomization	Ischemic Heart Disease	Fisher	0.778483	1
Randomization	Chronic Liver Disease	Chi-squared	0.813951	1
Randomization	Drugs Used In Diabetes	Chi-squared	0.151655	1
Randomization	Diuretics	Chi-squared	0.69483	1
Randomization	Beta Blocking Agents	Chi-squared	0.475822	1
Randomization	Calcium Channel Blockers	Chi-squared	1	1
Randomization	Agents Acting On The Renin-Angiotensin System	Chi-squared	0.991605	1
Randomization	Lipid Modifying Agents	Chi-squared	0.164446	1
Randomization	Antibacterials For Systemic Use	Chi-squared	0.041384	1
Randomization	Drugs For Obstructive Airway Diseases	Chi-squared	1	1
Randomization	Immunosuppressants	Chi-squared	0.018148	1
Randomization	Antiinflammatory And Antirheumatic Products	Chi-squared	0.848937	1
Randomization	Antiepileptics	Chi-squared	0.731333	1
Randomization	Psycholeptics	Chi-squared	0.070939	1
Randomization	Antidepressants	Chi-squared	0.416539	1
Randomization	Age	Chi-squared	0.077455	1
Randomization	Ethnicity	Chi-squared	0.295609	1
Randomization	Race	Chi-squared	0.130266	1
Blinding	Lesion Of Liver	Chi-squared	4.32E-06	0.001762
Blinding	Malignant Tumor Of Breast	Chi-squared	8.06E-06	0.003288
Blinding	Opioids	Chi-squared	4.37E-05	0.017821
Blinding	Diabetes Mellitus	Chi-squared	0.000438	0.178565
Blinding	Drugs Used In Diabetes	Chi-squared	0.001414	0.576988
Blinding	Female	Chi-squared	0.052889	1
Blinding	Osteoarthritis	Chi-squared	0.62259	1

© 2021 Rogers JR et al. *JAMA Network Open*.

Trial Characteristic	Covariate Label	Statistical Test	P-value	Adjusted P-value
Blinding	Urinary Tract Infectious Disease	Chi-squared	0.138113	1
Blinding	Viral Hepatitis C	Fisher	0.388463	1
Blinding	Malignant Tumor Of Urinary Bladder	Chi-squared	0.00285	1
Blinding	Primary Malignant Neoplasm Of Prostate	Chi-squared	0.74356	1
Blinding	Chronic Obstructive Lung Disease	Chi-squared	0.440693	1
Blinding	Pneumonia	Chi-squared	0.556525	1
Blinding	Atrial Fibrillation	Chi-squared	0.109167	1
Blinding	Heart Failure	Fisher	0.604363	1
Blinding	Hypertensive Disorder	Chi-squared	0.010696	1
Blinding	Coronary Arteriosclerosis	Chi-squared	1	1
Blinding	Gastroesophageal Reflux Disease	Chi-squared	0.537719	1
Blinding	Peripheral Vascular Disease	Fisher	0.597994	1
Blinding	Heart Disease	Chi-squared	0.917357	1
Blinding	Cerebrovascular Disease	Fisher	0.206704	1
Blinding	Malignant Lymphoma	Chi-squared	0.557516	1
Blinding	Hyperlipidemia	Chi-squared	0.079765	1
Blinding	Obesity	Fisher	0.768384	1
Blinding	Depressive Disorder	Chi-squared	1	1
Blinding	Malignant Tumor Of Lung	Chi-squared	0.006203	1
Blinding	Venous Thrombosis	Chi-squared	0.948776	1
Blinding	Acute Respiratory Disease	Chi-squared	0.455962	1
Blinding	Renal Impairment	Chi-squared	1	1
Blinding	Hematologic Neoplasm	Chi-squared	0.596932	1
Blinding	Visual System Disorder	Chi-squared	0.773512	1
Blinding	Ischemic Heart Disease	Fisher	0.035819	1
Blinding	Chronic Liver Disease	Fisher	0.175808	1
Blinding	Drugs For Acid Related Disorders	Chi-squared	0.003107	1
Blinding	Antithrombotic Agents	Chi-squared	0.162037	1
Blinding	Antineoplastic Agents	Chi-squared	0.575874	1
Blinding	Diuretics	Chi-squared	0.071544	1
Blinding	Beta Blocking Agents	Chi-squared	0.598002	1
Blinding	Calcium Channel Blockers	Chi-squared	0.066918	1
Blinding	Agents Acting On The Renin-Angiotensin System	Chi-squared	0.044884	1
Blinding	Lipid Modifying Agents	Chi-squared	0.034155	1
Blinding	Antibacterials For Systemic Use	Chi-squared	0.795254	1
Blinding	Drugs For Obstructive Airway Diseases	Chi-squared	0.894329	1
Blinding	Immunosuppressants	Chi-squared	0.279815	1
Blinding	Antiinflammatory And Antirheumatic Products	Chi-squared	0.388733	1

© 2021 Rogers JR et al. *JAMA Network Open*.

Trial Characteristic	Covariate Label	Statistical Test	P-value	Adjusted P-value
Blinding	Antiepileptics	Chi-squared	0.134705	1
Blinding	Psycholeptics	Chi-squared	0.00537	1
Blinding	Antidepressants	Chi-squared	0.976538	1
Blinding	Age	Chi-squared	0.007433	1
Blinding	Ethnicity	Chi-squared	0.250476	1
Blinding	Race	Fisher	0.059717	1
Industry Sponsor	Age	Chi-squared	1.55E-20	6.31E-18
Industry Sponsor	Malignant Tumor Of Urinary Bladder	Chi-squared	0.000262	0.107093
Industry Sponsor	Race	Fisher	0.0005	0.20397
Industry Sponsor	Female	Chi-squared	0.444301	1
Industry Sponsor	Osteoarthritis	Chi-squared	0.031027	1
Industry Sponsor	Urinary Tract Infectious Disease	Chi-squared	0.723275	1
Industry Sponsor	Viral Hepatitis C	Fisher	0.699474	1
Industry Sponsor	Primary Malignant Neoplasm Of Prostate	Chi-squared	0.004887	1
Industry Sponsor	Diabetes Mellitus	Chi-squared	0.890919	1
Industry Sponsor	Chronic Obstructive Lung Disease	Chi-squared	0.897024	1
Industry Sponsor	Pneumonia	Chi-squared	1	1
Industry Sponsor	Atrial Fibrillation	Chi-squared	0.834855	1
Industry Sponsor	Heart Failure	Chi-squared	0.390361	1
Industry Sponsor	Hypertensive Disorder	Chi-squared	0.608764	1
Industry Sponsor	Coronary Arteriosclerosis	Chi-squared	0.922709	1
Industry Sponsor	Gastroesophageal Reflux Disease	Chi-squared	0.010372	1
Industry Sponsor	Peripheral Vascular Disease	Fisher	1	1
Industry Sponsor	Heart Disease	Chi-squared	0.20268	1
Industry Sponsor	Cerebrovascular Disease	Chi-squared	0.604021	1
Industry Sponsor	Malignant Lymphoma	Chi-squared	0.02218	1
Industry Sponsor	Hyperlipidemia	Chi-squared	0.744417	1
Industry Sponsor	Obesity	Fisher	0.010635	1
Industry Sponsor	Depressive Disorder	Chi-squared	0.81981	1
Industry Sponsor	Malignant Tumor Of Lung	Chi-squared	0.089254	1
Industry Sponsor	Venous Thrombosis	Chi-squared	0.864715	1
Industry Sponsor	Acute Respiratory Disease	Chi-squared	0.289422	1
Industry Sponsor	Renal Impairment	Chi-squared	1	1
Industry Sponsor	Hematologic Neoplasm	Chi-squared	0.623385	1
Industry Sponsor	Lesion Of Liver	Chi-squared	0.040027	1
Industry Sponsor	Malignant Tumor Of Breast	Chi-squared	0.1008	1
Industry Sponsor	Visual System Disorder	Chi-squared	0.883959	1
Industry Sponsor	Ischemic Heart Disease	Fisher	0.484093	1
Industry Sponsor	Chronic Liver Disease	Fisher	0.610786	1
Industry Sponsor	Drugs For Acid Related Disorders	Chi-squared	0.400269	1

© 2021 Rogers JR et al. *JAMA Network Open*.

Trial Characteristic	Covariate Label	Statistical Test	P-value	Adjusted P-value
Industry Sponsor	Drugs Used In Diabetes	Chi-squared	0.191071	1
Industry Sponsor	Antithrombotic Agents	Chi-squared	0.570113	1
Industry Sponsor	Antineoplastic Agents	Chi-squared	0.88576	1
Industry Sponsor	Diuretics	Chi-squared	0.151648	1
Industry Sponsor	Beta Blocking Agents	Chi-squared	0.014913	1
Industry Sponsor	Calcium Channel Blockers	Chi-squared	0.087475	1
Industry Sponsor	Agents Acting On The Renin-Angiotensin System	Chi-squared	0.008666	1
Industry Sponsor	Lipid Modifying Agents	Chi-squared	0.003341	1
Industry Sponsor	Antibacterials For Systemic Use	Chi-squared	0.452407	1
Industry Sponsor	Drugs For Obstructive Airway Diseases	Chi-squared	0.712349	1
Industry Sponsor	Immunosuppressants	Chi-squared	0.391082	1
Industry Sponsor	Antiinflammatory And Antirheumatic Products	Chi-squared	0.564101	1
Industry Sponsor	Opioids	Chi-squared	0.750132	1
Industry Sponsor	Antiepileptics	Chi-squared	0.077607	1
Industry Sponsor	Psycholeptics	Chi-squared	0.41952	1
Industry Sponsor	Antidepressants	Chi-squared	0.324029	1
Industry Sponsor	Ethnicity	Chi-squared	0.020853	1
Use of DMC	Malignant Tumor Of Lung	Chi-squared	3.5E-11	1.43E-08
Use of DMC	Age	Chi-squared	1.25E-07	5.12E-05
Use of DMC	Malignant Tumor Of Urinary Bladder	Chi-squared	1.84E-07	7.5E-05
Use of DMC	Malignant Lymphoma	Chi-squared	1.49E-06	0.000609
Use of DMC	Primary Malignant Neoplasm Of Prostate	Chi-squared	8.82E-05	0.035977
Use of DMC	Malignant Tumor Of Breast	Chi-squared	0.001123	0.45821
Use of DMC	Urinary Tract Infectious Disease	Chi-squared	0.002194	0.895008
Use of DMC	Female	Chi-squared	0.019426	1
Use of DMC	Osteoarthritis	Chi-squared	0.540933	1
Use of DMC	Viral Hepatitis C	Fisher	0.028466	1
Use of DMC	Diabetes Mellitus	Chi-squared	0.070385	1
Use of DMC	Chronic Obstructive Lung Disease	Chi-squared	0.912351	1
Use of DMC	Pneumonia	Chi-squared	0.801268	1
Use of DMC	Atrial Fibrillation	Chi-squared	1	1
Use of DMC	Heart Failure	Chi-squared	0.200013	1
Use of DMC	Hypertensive Disorder	Chi-squared	0.325746	1
Use of DMC	Coronary Arteriosclerosis	Chi-squared	0.373798	1
Use of DMC	Gastroesophageal Reflux Disease	Chi-squared	0.104816	1
Use of DMC	Peripheral Vascular Disease	Fisher	0.704347	1
Use of DMC	Heart Disease	Chi-squared	0.521815	1
Use of DMC	Cerebrovascular Disease	Chi-squared	1	1

© 2021 Rogers JR et al. *JAMA Network Open*.

Trial Characteristic	Covariate Label	Statistical Test	P-value	Adjusted P-value
Use of DMC	Hyperlipidemia	Chi-squared	0.250091	1
Use of DMC	Obesity	Chi-squared	0.14657	1
Use of DMC	Depressive Disorder	Chi-squared	0.229761	1
Use of DMC	Venous Thrombosis	Chi-squared	0.846257	1
Use of DMC	Acute Respiratory Disease	Chi-squared	0.765959	1
Use of DMC	Renal Impairment	Chi-squared	0.256169	1
Use of DMC	Hematologic Neoplasm	Chi-squared	0.037467	1
Use of DMC	Lesion Of Liver	Chi-squared	0.075481	1
Use of DMC	Visual System Disorder	Chi-squared	0.057565	1
Use of DMC	Ischemic Heart Disease	Chi-squared	0.607644	1
Use of DMC	Chronic Liver Disease	Chi-squared	0.030978	1
Use of DMC	Drugs For Acid Related Disorders	Chi-squared	0.989767	1
Use of DMC	Drugs Used In Diabetes	Chi-squared	0.9055	1
Use of DMC	Antithrombotic Agents	Chi-squared	0.269299	1
Use of DMC	Antineoplastic Agents	Chi-squared	0.126302	1
Use of DMC	Diuretics	Chi-squared	0.025522	1
Use of DMC	Beta Blocking Agents	Chi-squared	0.003568	1
Use of DMC	Calcium Channel Blockers	Chi-squared	0.802358	1
Use of DMC	Agents Acting On The Renin-Angiotensin System	Chi-squared	1	1
Use of DMC	Lipid Modifying Agents	Chi-squared	0.718952	1
Use of DMC	Antibacterials For Systemic Use	Chi-squared	0.966589	1
Use of DMC	Drugs For Obstructive Airway Diseases	Chi-squared	0.763495	1
Use of DMC	Immunosuppressants	Chi-squared	0.416909	1
Use of DMC	Antiinflammatory And Antirheumatic Products	Chi-squared	0.012481	1
Use of DMC	Opioids	Chi-squared	0.064361	1
Use of DMC	Antiepileptics	Chi-squared	0.05392	1
Use of DMC	Psycholeptics	Chi-squared	0.062433	1
Use of DMC	Antidepressants	Chi-squared	0.017734	1
Use of DMC	Ethnicity	Chi-squared	0.695258	1
Use of DMC	Race	Chi-squared	0.599072	1
Multi-site Trial	Malignant Tumor Of Urinary Bladder	Chi-squared	5.15E-55	2.1E-52
Multi-site Trial	Urinary Tract Infectious Disease	Chi-squared	2.4E-11	9.8E-09
Multi-site Trial	Age	Chi-squared	1.04E-05	0.004245
Multi-site Trial	Hematologic Neoplasm	Chi-squared	4.25E-05	0.017339
Multi-site Trial	Hyperlipidemia	Chi-squared	0.000129	0.052477
Multi-site Trial	Hypertensive Disorder	Chi-squared	0.000358	0.146031
Multi-site Trial	Female	Chi-squared	0.019362	1
Multi-site Trial	Osteoarthritis	Chi-squared	0.473869	1

© 2021 Rogers JR et al. *JAMA Network Open*.

Trial Characteristic	Covariate Label	Statistical Test	P-value	Adjusted P-value
Multi-site Trial	Viral Hepatitis C	Fisher	0.22668	1
Multi-site Trial	Primary Malignant Neoplasm Of Prostate	Chi-squared	0.795592	1
Multi-site Trial	Diabetes Mellitus	Chi-squared	0.896666	1
Multi-site Trial	Chronic Obstructive Lung Disease	Chi-squared	0.619113	1
Multi-site Trial	Pneumonia	Chi-squared	0.463987	1
Multi-site Trial	Atrial Fibrillation	Chi-squared	1	1
Multi-site Trial	Heart Failure	Fisher	0.615283	1
Multi-site Trial	Coronary Arteriosclerosis	Chi-squared	0.203976	1
Multi-site Trial	Gastroesophageal Reflux Disease	Chi-squared	0.185973	1
Multi-site Trial	Peripheral Vascular Disease	Fisher	0.060437	1
Multi-site Trial	Heart Disease	Chi-squared	0.006849	1
Multi-site Trial	Cerebrovascular Disease	Chi-squared	0.499276	1
Multi-site Trial	Malignant Lymphoma	Chi-squared	0.727931	1
Multi-site Trial	Obesity	Fisher	0.782575	1
Multi-site Trial	Depressive Disorder	Chi-squared	0.66809	1
Multi-site Trial	Malignant Tumor Of Lung	Chi-squared	0.376835	1
Multi-site Trial	Venous Thrombosis	Chi-squared	0.285124	1
Multi-site Trial	Acute Respiratory Disease	Chi-squared	1	1
Multi-site Trial	Renal Impairment	Chi-squared	0.597569	1
Multi-site Trial	Lesion Of Liver	Chi-squared	0.131223	1
Multi-site Trial	Malignant Tumor Of Breast	Chi-squared	0.353535	1
Multi-site Trial	Visual System Disorder	Chi-squared	0.563949	1
Multi-site Trial	Ischemic Heart Disease	Fisher	1	1
Multi-site Trial	Chronic Liver Disease	Fisher	0.067919	1
Multi-site Trial	Drugs For Acid Related Disorders	Chi-squared	0.561603	1
Multi-site Trial	Drugs Used In Diabetes	Chi-squared	0.304791	1
Multi-site Trial	Antithrombotic Agents	Chi-squared	0.998148	1
Multi-site Trial	Antineoplastic Agents	Chi-squared	0.074468	1
Multi-site Trial	Diuretics	Chi-squared	0.540291	1
Multi-site Trial	Beta Blocking Agents	Chi-squared	0.496807	1
Multi-site Trial	Calcium Channel Blockers	Chi-squared	0.522113	1
Multi-site Trial	Agents Acting On The Renin-Angiotensin System	Chi-squared	0.030889	1
Multi-site Trial	Lipid Modifying Agents	Chi-squared	0.075553	1
Multi-site Trial	Antibacterials For Systemic Use	Chi-squared	0.567173	1
Multi-site Trial	Drugs For Obstructive Airway Diseases	Chi-squared	1	1
Multi-site Trial	Immunosuppressants	Chi-squared	0.094013	1
Multi-site Trial	Antiinflammatory And Antirheumatic Products	Chi-squared	1	1
Multi-site Trial	Opioids	Chi-squared	0.536102	1

© 2021 Rogers JR et al. *JAMA Network Open*.

Trial Characteristic	Covariate Label	Statistical Test	P-value	Adjusted P-value
Multi-site Trial	Antiepileptics	Chi-squared	0.016369	1
Multi-site Trial	Psycholeptics	Chi-squared	0.011384	1
Multi-site Trial	Antidepressants	Chi-squared	0.507011	1
Multi-site Trial	Ethnicity	Chi-squared	0.03778	1
Multi-site Trial	Race	Fisher	0.021296	1
Overall Enrollment	Malignant Tumor Of Urinary Bladder	Chi-squared	5.96E-35	2.43E-32
Overall Enrollment	Age	Chi-squared	6.47E-16	2.64E-13
Overall Enrollment	Urinary Tract Infectious Disease	Chi-squared	9.28E-07	0.000379
Overall Enrollment	Viral Hepatitis C	Fisher	0.000221	0.090314
Overall Enrollment	Hematologic Neoplasm	Chi-squared	0.000237	0.096525
Overall Enrollment	Lesion Of Liver	Chi-squared	0.000396	0.161456
Overall Enrollment	Primary Malignant Neoplasm Of Prostate	Chi-squared	0.000551	0.224715
Overall Enrollment	Agents Acting On The Renin-Angiotensin System	Chi-squared	0.000628	0.25602
Overall Enrollment	Hypertensive Disorder	Chi-squared	0.001678	0.684744
Overall Enrollment	Female	Chi-squared	0.022659	1
Overall Enrollment	Osteoarthritis	Chi-squared	0.537939	1
Overall Enrollment	Diabetes Mellitus	Chi-squared	0.067371	1
Overall Enrollment	Chronic Obstructive Lung Disease	Chi-squared	0.209338	1
Overall Enrollment	Pneumonia	Chi-squared	0.858945	1
Overall Enrollment	Atrial Fibrillation	Chi-squared	0.663069	1
Overall Enrollment	Heart Failure	Chi-squared	0.043112	1
Overall Enrollment	Coronary Arteriosclerosis	Chi-squared	0.226839	1
Overall Enrollment	Gastroesophageal Reflux Disease	Chi-squared	0.265194	1
Overall Enrollment	Peripheral Vascular Disease	Fisher	0.217147	1
Overall Enrollment	Heart Disease	Chi-squared	0.002462	1
Overall Enrollment	Cerebrovascular Disease	Chi-squared	0.781042	1
Overall Enrollment	Malignant Lymphoma	Chi-squared	0.609655	1
Overall Enrollment	Hyperlipidemia	Chi-squared	0.01415	1
Overall Enrollment	Obesity	Fisher	0.302081	1
Overall Enrollment	Depressive Disorder	Chi-squared	0.7832	1
Overall Enrollment	Malignant Tumor Of Lung	Chi-squared	0.096457	1
Overall Enrollment	Venous Thrombosis	Chi-squared	0.743548	1
Overall Enrollment	Acute Respiratory Disease	Chi-squared	0.950893	1
Overall Enrollment	Renal Impairment	Chi-squared	0.229048	1
Overall Enrollment	Malignant Tumor Of Breast	Chi-squared	0.075102	1
Overall Enrollment	Visual System Disorder	Chi-squared	0.671687	1
Overall Enrollment	Ischemic Heart Disease	Fisher	0.469949	1
Overall Enrollment	Chronic Liver Disease	Chi-squared	0.015389	1
Overall Enrollment	Drugs For Acid Related Disorders	Chi-squared	0.453647	1

© 2021 Rogers JR et al. *JAMA Network Open*.

Trial Characteristic	Covariate Label	Statistical Test	P-value	Adjusted P-value
Overall Enrollment	Drugs Used In Diabetes	Chi-squared	0.229581	1
Overall Enrollment	Antithrombotic Agents	Chi-squared	0.655599	1
Overall Enrollment	Antineoplastic Agents	Chi-squared	0.260028	1
Overall Enrollment	Diuretics	Chi-squared	0.708954	1
Overall Enrollment	Beta Blocking Agents	Chi-squared	0.143726	1
Overall Enrollment	Calcium Channel Blockers	Chi-squared	0.48821	1
Overall Enrollment	Lipid Modifying Agents	Chi-squared	0.420891	1
Overall Enrollment	Antibacterials For Systemic Use	Chi-squared	0.977348	1
Overall Enrollment	Drugs For Obstructive Airway Diseases	Chi-squared	0.700206	1
Overall Enrollment	Immunosuppressants	Chi-squared	0.040223	1
Overall Enrollment	Antiinflammatory And Antirheumatic Products	Chi-squared	0.112126	1
Overall Enrollment	Opioids	Chi-squared	0.740024	1
Overall Enrollment	Antiepileptics	Chi-squared	0.045818	1
Overall Enrollment	Psycholeptics	Chi-squared	0.192702	1
Overall Enrollment	Antidepressants	Chi-squared	0.028621	1
Overall Enrollment	Ethnicity	Chi-squared	0.395825	1
Overall Enrollment	Race	Chi-squared	0.369372	1

© 2021 Rogers JR et al. *JAMA Network Open*.

eTable 4: Associations between trial participants' covariates and trial characteristics, disorder of digestive system trials

Trial Characteristic	Covariate Label	Statistical Test	P-value	Adjusted P-value
Phase	Malignant Neoplastic Disease	Chi-squared	3.67E-19	1.53E-16
Phase	Primary Malignant Neoplasm Of Prostate	Fisher	3.35E-16	1.39E-13
Phase	Chronic Liver Disease	Chi-squared	1.47E-15	6.13E-13
Phase	Antithrombotic Agents	Chi-squared	1.59E-15	6.61E-13
Phase	Viral Hepatitis C	Chi-squared	2.48E-15	1.03E-12
Phase	Lesion Of Liver	Chi-squared	1.86E-13	7.74E-11
Phase	Opioids	Chi-squared	1.14E-12	4.76E-10
Phase	Psycholeptics	Chi-squared	3.03E-09	1.26E-06
Phase	Antibacterials For Systemic Use	Chi-squared	6.21E-09	2.58E-06
Phase	Immunosuppressants	Chi-squared	2.41E-08	1E-05
Phase	Calcium Channel Blockers	Chi-squared	2.1E-07	8.75E-05
Phase	Ethnicity	Fisher	8.86E-07	0.000369
Phase	Drugs For Acid Related Disorders	Chi-squared	1.22E-06	0.000507
Phase	Beta Blocking Agents	Chi-squared	1.38E-06	0.000573
Phase	Diuretics	Chi-squared	1.4E-06	0.000584
Phase	Race	Fisher	1.61E-06	0.000671
Phase	Age	Chi-squared	6.77E-06	0.002817
Phase	Hypertensive Disorder	Chi-squared	8.35E-06	0.003473
Phase	Renal Impairment	Fisher	6.72E-05	0.027967
Phase	Drugs Used In Diabetes	Chi-squared	0.000122	0.050826
Phase	Osteoarthritis	Fisher	0.000162	0.067196
Phase	Heart Disease	Chi-squared	0.000182	0.075537
Phase	Malignant Tumor Of Lung	Fisher	0.000293	0.121986
Phase	Female	Chi-squared	0.001274	0.529811
Phase	Antineoplastic Agents	Chi-squared	0.00163	0.677966
Phase	Chronic Obstructive Lung Disease	Fisher	0.001724	0.717063
Phase	Urinary Tract Infectious Disease	Fisher	0.810025	1
Phase	Malignant Tumor Of Urinary Bladder	Fisher	0.363649	1
Phase	Diabetes Mellitus	Chi-squared	0.026994	1
Phase	Pneumonia	Fisher	0.054381	1
Phase	Atrial Fibrillation	Fisher	0.554021	1
Phase	Heart Failure	Fisher	0.306777	1
Phase	Coronary Arteriosclerosis	Fisher	0.223732	1
Phase	Gastroesophageal Reflux Disease	Fisher	0.008972	1
Phase	Peripheral Vascular Disease	Fisher	0.804251	1
Phase	Cerebrovascular Disease	Fisher	0.039989	1
Phase	Malignant Lymphoma	Fisher	1	1

© 2021 Rogers JR et al. *JAMA Network Open*.

Trial Characteristic	Covariate Label	Statistical Test	P-value	Adjusted P-value
Phase	Hyperlipidemia	Fisher	0.06217	1
Phase	Obesity	Fisher	0.159926	1
Phase	Depressive Disorder	Fisher	0.021504	1
Phase	Venous Thrombosis	Fisher	0.004412	1
Phase	Acute Respiratory Disease	Fisher	0.01849	1
Phase	Hematologic Neoplasm	Fisher	0.003085	1
Phase	Malignant Tumor Of Breast	Fisher	0.679255	1
Phase	Visual System Disorder	Fisher	0.150199	1
Phase	Ischemic Heart Disease	Fisher	0.869501	1
Phase	Agents Acting On The Renin-Angiotensin System	Chi-squared	0.015633	1
Phase	Lipid Modifying Agents	Fisher	0.003777	1
Phase	Drugs For Obstructive Airway Diseases	Fisher	0.546999	1
Phase	Antiinflammatory And Antirheumatic Products	Chi-squared	0.006268	1
Phase	Antiepileptics	Fisher	0.144161	1
Phase	Antidepressants	Chi-squared	0.003513	1
Number of Treatment Arms	Opioids	Chi-squared	1.08E-07	4.51E-05
Number of Treatment Arms	Antithrombotic Agents	Chi-squared	8.47E-06	0.003522
Number of Treatment Arms	Malignant Neoplastic Disease	Chi-squared	4.09E-05	0.017025
Number of Treatment Arms	Antineoplastic Agents	Chi-squared	6.2E-05	0.025793
Number of Treatment Arms	Chronic Liver Disease	Chi-squared	8.02E-05	0.03335
Number of Treatment Arms	Immunosuppressants	Chi-squared	0.000125	0.052206
Number of Treatment Arms	Primary Malignant Neoplasm Of Prostate	Fisher	0.000145	0.060412
Number of Treatment Arms	Malignant Tumor Of Lung	Fisher	0.000177	0.07367
Number of Treatment Arms	Antibacterials For Systemic Use	Chi-squared	0.000201	0.083632
Number of Treatment Arms	Drugs For Acid Related Disorders	Chi-squared	0.000628	0.261227
Number of Treatment Arms	Viral Hepatitis C	Chi-squared	0.00109	0.453579
Number of Treatment Arms	Female	Chi-squared	0.008162	1
Number of Treatment Arms	Osteoarthritis	Fisher	0.236819	1
Number of Treatment Arms	Urinary Tract Infectious Disease	Fisher	0.064568	1

© 2021 Rogers JR et al. *JAMA Network Open*.

Trial Characteristic	Covariate Label	Statistical Test	P-value	Adjusted P-value
Number of Treatment Arms	Malignant Tumor Of Urinary Bladder	Fisher	0.425584	1
Number of Treatment Arms	Diabetes Mellitus	Chi-squared	0.265412	1
Number of Treatment Arms	Chronic Obstructive Lung Disease	Fisher	0.009529	1
Number of Treatment Arms	Pneumonia	Fisher	1	1
Number of Treatment Arms	Atrial Fibrillation	Fisher	0.765604	1
Number of Treatment Arms	Heart Failure	Fisher	0.144626	1
Number of Treatment Arms	Hypertensive Disorder	Chi-squared	0.011295	1
Number of Treatment Arms	Coronary Arteriosclerosis	Fisher	0.207165	1
Number of Treatment Arms	Gastroesophageal Reflux Disease	Fisher	0.374168	1
Number of Treatment Arms	Peripheral Vascular Disease	Fisher	0.280275	1
Number of Treatment Arms	Heart Disease	Chi-squared	0.008359	1
Number of Treatment Arms	Cerebrovascular Disease	Fisher	0.185446	1
Number of Treatment Arms	Malignant Lymphoma	Fisher	1	1
Number of Treatment Arms	Hyperlipidemia	Fisher	0.473112	1
Number of Treatment Arms	Obesity	Fisher	0.100525	1
Number of Treatment Arms	Depressive Disorder	Fisher	0.586486	1
Number of Treatment Arms	Venous Thrombosis	Fisher	0.220979	1
Number of Treatment Arms	Acute Respiratory Disease	Fisher	0.254811	1
Number of Treatment Arms	Renal Impairment	Chi-squared	0.274196	1
Number of Treatment Arms	Hematologic Neoplasm	Fisher	0.692567	1
Number of Treatment Arms	Lesion Of Liver	Chi-squared	0.003492	1
Number of Treatment Arms	Malignant Tumor Of Breast	Fisher	0.028779	1
Number of Treatment Arms	Visual System Disorder	Fisher	0.826543	1
Number of Treatment Arms	Ischemic Heart Disease	Fisher	0.375256	1

© 2021 Rogers JR et al. *JAMA Network Open*.

Trial Characteristic	Covariate Label	Statistical Test	P-value	Adjusted P-value
Number of Treatment Arms	Drugs Used In Diabetes	Chi-squared	0.278931	1
Number of Treatment Arms	Diuretics	Chi-squared	0.0134	1
Number of Treatment Arms	Beta Blocking Agents	Chi-squared	0.779616	1
Number of Treatment Arms	Calcium Channel Blockers	Chi-squared	0.019986	1
Number of Treatment Arms	Agents Acting On The Renin-Angiotensin System	Chi-squared	0.523272	1
Number of Treatment Arms	Lipid Modifying Agents	Chi-squared	0.32835	1
Number of Treatment Arms	Drugs For Obstructive Airway Diseases	Chi-squared	0.757989	1
Number of Treatment Arms	Antiinflammatory And Antirheumatic Products	Chi-squared	0.167528	1
Number of Treatment Arms	Antiepileptics	Fisher	0.72375	1
Number of Treatment Arms	Psycholeptics	Chi-squared	0.148737	1
Number of Treatment Arms	Antidepressants	Chi-squared	0.147957	1
Number of Treatment Arms	Age	Chi-squared	0.376318	1
Number of Treatment Arms	Ethnicity	Chi-squared	0.461762	1
Number of Treatment Arms	Race	Fisher	0.024535	1
Randomization	Primary Malignant Neoplasm Of Prostate	Chi-squared	1.7E-11	7.06E-09
Randomization	Calcium Channel Blockers	Chi-squared	1.56E-06	0.000649
Randomization	Female	Chi-squared	5.02E-05	0.02089
Randomization	Malignant Neoplastic Disease	Chi-squared	0.000129	0.053702
Randomization	Hypertensive Disorder	Chi-squared	0.000292	0.121511
Randomization	Malignant Tumor Of Lung	Chi-squared	0.001214	0.505189
Randomization	Osteoarthritis	Chi-squared	0.001967	0.818122
Randomization	Urinary Tract Infectious Disease	Fisher	1	1
Randomization	Viral Hepatitis C	Chi-squared	0.093572	1
Randomization	Malignant Tumor Of Urinary Bladder	Fisher	0.491706	1
Randomization	Diabetes Mellitus	Chi-squared	0.637158	1
Randomization	Chronic Obstructive Lung Disease	Fisher	0.083982	1
Randomization	Pneumonia	Fisher	0.730018	1
Randomization	Atrial Fibrillation	Fisher	1	1
Randomization	Heart Failure	Fisher	0.77064	1
Randomization	Coronary Arteriosclerosis	Chi-squared	0.694943	1
Randomization	Gastroesophageal Reflux Disease	Chi-squared	0.518746	1

© 2021 Rogers JR et al. *JAMA Network Open*.

Trial Characteristic	Covariate Label	Statistical Test	P-value	Adjusted P-value
Randomization	Peripheral Vascular Disease	Fisher	1	1
Randomization	Heart Disease	Chi-squared	0.639282	1
Randomization	Cerebrovascular Disease	Fisher	1	1
Randomization	Malignant Lymphoma	Fisher	1	1
Randomization	Hyperlipidemia	Chi-squared	0.406558	1
Randomization	Obesity	Fisher	1	1
Randomization	Depressive Disorder	Chi-squared	0.584077	1
Randomization	Venous Thrombosis	Fisher	0.100006	1
Randomization	Acute Respiratory Disease	Chi-squared	0.694011	1
Randomization	Renal Impairment	Chi-squared	0.011563	1
Randomization	Hematologic Neoplasm	Fisher	0.020357	1
Randomization	Lesion Of Liver	Chi-squared	0.035893	1
Randomization	Malignant Tumor Of Breast	Fisher	0.19852	1
Randomization	Visual System Disorder	Chi-squared	1	1
Randomization	Ischemic Heart Disease	Fisher	0.581427	1
Randomization	Chronic Liver Disease	Chi-squared	0.035893	1
Randomization	Drugs For Acid Related Disorders	Chi-squared	0.902348	1
Randomization	Drugs Used In Diabetes	Chi-squared	0.81229	1
Randomization	Antithrombotic Agents	Chi-squared	0.42676	1
Randomization	Antineoplastic Agents	Chi-squared	0.02726	1
Randomization	Diuretics	Chi-squared	0.137767	1
Randomization	Beta Blocking Agents	Chi-squared	0.903942	1
Randomization	Agents Acting On The Renin-Angiotensin System	Chi-squared	0.384356	1
Randomization	Lipid Modifying Agents	Chi-squared	0.669971	1
Randomization	Antibacterials For Systemic Use	Chi-squared	0.872466	1
Randomization	Drugs For Obstructive Airway Diseases	Chi-squared	0.617325	1
Randomization	Immunosuppressants	Chi-squared	0.004667	1
Randomization	Antiinflammatory And Antirheumatic Products	Chi-squared	0.409323	1
Randomization	Opioids	Chi-squared	0.407112	1
Randomization	Antiepileptics	Chi-squared	0.671909	1
Randomization	Psycholeptics	Chi-squared	0.04274	1
Randomization	Antidepressants	Chi-squared	0.732096	1
Randomization	Age	Chi-squared	0.257761	1
Randomization	Ethnicity	Chi-squared	0.247453	1
Randomization	Race	Fisher	0.088668	1
Blinding	Lesion Of Liver	Chi-squared	0.001207	0.502141
Blinding	Drugs For Acid Related Disorders	Chi-squared	0.001207	0.502141
Blinding	Female	Chi-squared	0.145276	1

© 2021 Rogers JR et al. *JAMA Network Open*.

Trial Characteristic	Covariate Label	Statistical Test	P-value	Adjusted P-value
Blinding	Osteoarthritis	Chi-squared	0.828869	1
Blinding	Urinary Tract Infectious Disease	Fisher	0.502473	1
Blinding	Viral Hepatitis C	Chi-squared	0.130285	1
Blinding	Malignant Tumor Of Urinary Bladder	Fisher	0.546145	1
Blinding	Primary Malignant Neoplasm Of Prostate	Chi-squared	0.006249	1
Blinding	Diabetes Mellitus	Chi-squared	0.509031	1
Blinding	Chronic Obstructive Lung Disease	Chi-squared	0.577615	1
Blinding	Pneumonia	Fisher	0.323103	1
Blinding	Atrial Fibrillation	Fisher	0.198468	1
Blinding	Heart Failure	Chi-squared	0.198412	1
Blinding	Hypertensive Disorder	Chi-squared	0.262624	1
Blinding	Coronary Arteriosclerosis	Chi-squared	0.776728	1
Blinding	Gastroesophageal Reflux Disease	Chi-squared	0.449662	1
Blinding	Peripheral Vascular Disease	Fisher	0.275369	1
Blinding	Heart Disease	Chi-squared	0.0067	1
Blinding	Cerebrovascular Disease	Fisher	0.05271	1
Blinding	Malignant Lymphoma	Fisher	1	1
Blinding	Hyperlipidemia	Chi-squared	0.049265	1
Blinding	Obesity	Fisher	0.771582	1
Blinding	Depressive Disorder	Chi-squared	1	1
Blinding	Malignant Tumor Of Lung	Chi-squared	0.189973	1
Blinding	Malignant Neoplastic Disease	Chi-squared	0.030606	1
Blinding	Venous Thrombosis	Chi-squared	0.395519	1
Blinding	Acute Respiratory Disease	Chi-squared	0.179131	1
Blinding	Renal Impairment	Chi-squared	1	1
Blinding	Hematologic Neoplasm	Chi-squared	0.601953	1
Blinding	Malignant Tumor Of Breast	Fisher	0.55397	1
Blinding	Visual System Disorder	Chi-squared	0.28811	1
Blinding	Ischemic Heart Disease	Fisher	0.121081	1
Blinding	Chronic Liver Disease	Chi-squared	0.221277	1
Blinding	Drugs Used In Diabetes	Chi-squared	0.009693	1
Blinding	Antithrombotic Agents	Chi-squared	0.70491	1
Blinding	Antineoplastic Agents	Chi-squared	0.471631	1
Blinding	Diuretics	Chi-squared	0.067078	1
Blinding	Beta Blocking Agents	Chi-squared	0.090293	1
Blinding	Calcium Channel Blockers	Chi-squared	0.252005	1
Blinding	Agents Acting On The Renin-Angiotensin System	Chi-squared	0.422436	1
Blinding	Lipid Modifying Agents	Chi-squared	0.110606	1
Blinding	Antibacterials For Systemic Use	Chi-squared	0.889105	1

© 2021 Rogers JR et al. *JAMA Network Open*.

Trial Characteristic	Covariate Label	Statistical Test	P-value	Adjusted P-value
Blinding	Drugs For Obstructive Airway Diseases	Chi-squared	1	1
Blinding	Immunosuppressants	Chi-squared	0.322379	1
Blinding	Antiinflammatory And Antirheumatic Products	Chi-squared	0.05648	1
Blinding	Opioids	Chi-squared	0.997364	1
Blinding	Antiepileptics	Chi-squared	0.419609	1
Blinding	Psycholeptics	Chi-squared	0.219167	1
Blinding	Antidepressants	Chi-squared	0.58167	1
Blinding	Age	Chi-squared	0.028407	1
Blinding	Ethnicity	Chi-squared	0.008404	1
Blinding	Race	Fisher	0.008109	1
Industry Sponsor	Antithrombotic Agents	Chi-squared	2.75E-20	1.14E-17
Industry Sponsor	Opioids	Chi-squared	7.07E-17	2.94E-14
Industry Sponsor	Antibacterials For Systemic Use	Chi-squared	4.78E-13	1.99E-10
Industry Sponsor	Immunosuppressants	Chi-squared	0.000872	0.362589
Industry Sponsor	Female	Chi-squared	0.953484	1
Industry Sponsor	Osteoarthritis	Chi-squared	0.713948	1
Industry Sponsor	Urinary Tract Infectious Disease	Fisher	0.458111	1
Industry Sponsor	Viral Hepatitis C	Chi-squared	0.043179	1
Industry Sponsor	Malignant Tumor Of Urinary Bladder	Fisher	1	1
Industry Sponsor	Primary Malignant Neoplasm Of Prostate	Chi-squared	0.048346	1
Industry Sponsor	Diabetes Mellitus	Chi-squared	0.580805	1
Industry Sponsor	Chronic Obstructive Lung Disease	Fisher	0.247146	1
Industry Sponsor	Pneumonia	Fisher	0.129631	1
Industry Sponsor	Atrial Fibrillation	Fisher	0.153036	1
Industry Sponsor	Heart Failure	Fisher	0.54209	1
Industry Sponsor	Hypertensive Disorder	Chi-squared	0.891588	1
Industry Sponsor	Coronary Arteriosclerosis	Chi-squared	0.192153	1
Industry Sponsor	Gastroesophageal Reflux Disease	Chi-squared	0.104439	1
Industry Sponsor	Peripheral Vascular Disease	Fisher	0.015623	1
Industry Sponsor	Heart Disease	Chi-squared	0.005101	1
Industry Sponsor	Cerebrovascular Disease	Fisher	0.153036	1
Industry Sponsor	Malignant Lymphoma	Fisher	0.441589	1
Industry Sponsor	Hyperlipidemia	Chi-squared	1	1
Industry Sponsor	Obesity	Fisher	0.100052	1
Industry Sponsor	Depressive Disorder	Chi-squared	1	1
Industry Sponsor	Malignant Tumor Of Lung	Chi-squared	0.677951	1
Industry Sponsor	Malignant Neoplastic Disease	Chi-squared	0.115857	1
Industry Sponsor	Venous Thrombosis	Fisher	0.77426	1
Industry Sponsor	Acute Respiratory Disease	Chi-squared	0.011843	1

© 2021 Rogers JR et al. *JAMA Network Open*.

Trial Characteristic	Covariate Label	Statistical Test	P-value	Adjusted P-value
Industry Sponsor	Renal Impairment	Chi-squared	0.119771	1
Industry Sponsor	Hematologic Neoplasm	Fisher	0.37503	1
Industry Sponsor	Lesion Of Liver	Chi-squared	0.62832	1
Industry Sponsor	Malignant Tumor Of Breast	Fisher	1	1
Industry Sponsor	Visual System Disorder	Chi-squared	1	1
Industry Sponsor	Ischemic Heart Disease	Fisher	1	1
Industry Sponsor	Chronic Liver Disease	Chi-squared	0.022399	1
Industry Sponsor	Drugs For Acid Related Disorders	Chi-squared	0.004267	1
Industry Sponsor	Drugs Used In Diabetes	Chi-squared	0.163783	1
Industry Sponsor	Antineoplastic Agents	Chi-squared	1	1
Industry Sponsor	Diuretics	Chi-squared	0.031631	1
Industry Sponsor	Beta Blocking Agents	Chi-squared	0.305201	1
Industry Sponsor	Calcium Channel Blockers	Chi-squared	0.454475	1
Industry Sponsor	Agents Acting On The Renin-Angiotensin System	Chi-squared	0.107003	1
Industry Sponsor	Lipid Modifying Agents	Chi-squared	0.043532	1
Industry Sponsor	Drugs For Obstructive Airway Diseases	Chi-squared	0.514092	1
Industry Sponsor	Antiinflammatory And Antirheumatic Products	Chi-squared	0.003943	1
Industry Sponsor	Antiepileptics	Chi-squared	1	1
Industry Sponsor	Psycholeptics	Chi-squared	1	1
Industry Sponsor	Antidepressants	Chi-squared	0.423677	1
Industry Sponsor	Age	Chi-squared	0.068101	1
Industry Sponsor	Ethnicity	Chi-squared	0.040697	1
Industry Sponsor	Race	Fisher	0.220022	1
Use of DMC	Immunosuppressants	Chi-squared	3.64E-09	1.51E-06
Use of DMC	Malignant Neoplastic Disease	Chi-squared	6.66E-08	2.77E-05
Use of DMC	Primary Malignant Neoplasm Of Prostate	Chi-squared	2.48E-07	0.000103
Use of DMC	Calcium Channel Blockers	Chi-squared	8.47E-06	0.003522
Use of DMC	Psycholeptics	Chi-squared	0.0001	0.04166
Use of DMC	Renal Impairment	Chi-squared	0.000694	0.288528
Use of DMC	Beta Blocking Agents	Chi-squared	0.000954	0.396834
Use of DMC	Age	Chi-squared	0.001101	0.458129
Use of DMC	Lesion Of Liver	Chi-squared	0.001428	0.594093
Use of DMC	Diuretics	Chi-squared	0.001473	0.612661
Use of DMC	Female	Chi-squared	0.068938	1
Use of DMC	Osteoarthritis	Chi-squared	0.003121	1
Use of DMC	Urinary Tract Infectious Disease	Fisher	0.280555	1
Use of DMC	Viral Hepatitis C	Chi-squared	0.155118	1
Use of DMC	Malignant Tumor Of Urinary Bladder	Fisher	0.104283	1

© 2021 Rogers JR et al. *JAMA Network Open*.

Trial Characteristic	Covariate Label	Statistical Test	P-value	Adjusted P-value
Use of DMC	Diabetes Mellitus	Chi-squared	1	1
Use of DMC	Chronic Obstructive Lung Disease	Chi-squared	0.204321	1
Use of DMC	Pneumonia	Fisher	0.082918	1
Use of DMC	Atrial Fibrillation	Fisher	0.185467	1
Use of DMC	Heart Failure	Fisher	0.781117	1
Use of DMC	Hypertensive Disorder	Chi-squared	0.025431	1
Use of DMC	Coronary Arteriosclerosis	Chi-squared	0.614678	1
Use of DMC	Gastroesophageal Reflux Disease	Chi-squared	1	1
Use of DMC	Peripheral Vascular Disease	Fisher	0.246161	1
Use of DMC	Heart Disease	Chi-squared	0.002602	1
Use of DMC	Cerebrovascular Disease	Fisher	0.006402	1
Use of DMC	Malignant Lymphoma	Fisher	0.543692	1
Use of DMC	Hyperlipidemia	Chi-squared	0.477606	1
Use of DMC	Obesity	Fisher	0.235634	1
Use of DMC	Depressive Disorder	Chi-squared	0.395938	1
Use of DMC	Malignant Tumor Of Lung	Chi-squared	0.023505	1
Use of DMC	Venous Thrombosis	Chi-squared	0.033503	1
Use of DMC	Acute Respiratory Disease	Chi-squared	0.039035	1
Use of DMC	Hematologic Neoplasm	Chi-squared	0.470718	1
Use of DMC	Malignant Tumor Of Breast	Fisher	1	1
Use of DMC	Visual System Disorder	Chi-squared	0.193437	1
Use of DMC	Ischemic Heart Disease	Fisher	1	1
Use of DMC	Chronic Liver Disease	Chi-squared	0.297568	1
Use of DMC	Drugs For Acid Related Disorders	Chi-squared	0.025445	1
Use of DMC	Drugs Used In Diabetes	Chi-squared	0.004177	1
Use of DMC	Antithrombotic Agents	Chi-squared	0.401499	1
Use of DMC	Antineoplastic Agents	Chi-squared	0.149888	1
Use of DMC	Agents Acting On The Renin-Angiotensin System	Chi-squared	0.910478	1
Use of DMC	Lipid Modifying Agents	Chi-squared	0.842206	1
Use of DMC	Antibacterials For Systemic Use	Chi-squared	0.226021	1
Use of DMC	Drugs For Obstructive Airway Diseases	Chi-squared	0.75232	1
Use of DMC	Antiinflammatory And Antirheumatic Products	Chi-squared	0.928905	1
Use of DMC	Opioids	Chi-squared	0.035016	1
Use of DMC	Antiepileptics	Chi-squared	0.058992	1
Use of DMC	Antidepressants	Chi-squared	0.196294	1
Use of DMC	Ethnicity	Chi-squared	0.203902	1
Use of DMC	Race	Fisher	0.081339	1
Multi-site Trial	Heart Disease	Chi-squared	2.49E-09	1.04E-06

© 2021 Rogers JR et al. *JAMA Network Open*.

Trial Characteristic	Covariate Label	Statistical Test	P-value	Adjusted P-value
Multi-site Trial	Immunosuppressants	Chi-squared	7.1E-08	2.95E-05
Multi-site Trial	Diuretics	Chi-squared	1.71E-07	7.11E-05
Multi-site Trial	Viral Hepatitis C	Chi-squared	6.68E-07	0.000278
Multi-site Trial	Chronic Liver Disease	Chi-squared	1.18E-06	0.000489
Multi-site Trial	Lesion Of Liver	Chi-squared	1.98E-05	0.008237
Multi-site Trial	Drugs Used In Diabetes	Chi-squared	0.000318	0.132144
Multi-site Trial	Beta Blocking Agents	Chi-squared	0.000415	0.172709
Multi-site Trial	Drugs For Acid Related Disorders	Chi-squared	0.00073	0.303565
Multi-site Trial	Acute Respiratory Disease	Fisher	0.001234	0.513354
Multi-site Trial	Renal Impairment	Chi-squared	0.001353	0.562676
Multi-site Trial	Pneumonia	Fisher	0.00237	0.98607
Multi-site Trial	Female	Chi-squared	0.34872	1
Multi-site Trial	Osteoarthritis	Fisher	0.606096	1
Multi-site Trial	Urinary Tract Infectious Disease	Fisher	0.654774	1
Multi-site Trial	Malignant Tumor Of Urinary Bladder	Fisher	1	1
Multi-site Trial	Primary Malignant Neoplasm Of Prostate	Fisher	0.143947	1
Multi-site Trial	Diabetes Mellitus	Chi-squared	0.723915	1
Multi-site Trial	Chronic Obstructive Lung Disease	Fisher	0.038508	1
Multi-site Trial	Atrial Fibrillation	Fisher	0.025107	1
Multi-site Trial	Heart Failure	Fisher	0.149316	1
Multi-site Trial	Hypertensive Disorder	Chi-squared	0.024327	1
Multi-site Trial	Coronary Arteriosclerosis	Fisher	0.010662	1
Multi-site Trial	Gastroesophageal Reflux Disease	Fisher	0.439718	1
Multi-site Trial	Peripheral Vascular Disease	Fisher	0.004804	1
Multi-site Trial	Cerebrovascular Disease	Fisher	0.025107	1
Multi-site Trial	Malignant Lymphoma	Fisher	0.313766	1
Multi-site Trial	Hyperlipidemia	Chi-squared	0.367985	1
Multi-site Trial	Obesity	Fisher	0.248738	1
Multi-site Trial	Depressive Disorder	Chi-squared	0.318596	1
Multi-site Trial	Malignant Tumor Of Lung	Fisher	0.767165	1
Multi-site Trial	Malignant Neoplastic Disease	Chi-squared	0.024325	1
Multi-site Trial	Venous Thrombosis	Fisher	0.184135	1
Multi-site Trial	Hematologic Neoplasm	Fisher	1	1
Multi-site Trial	Malignant Tumor Of Breast	Fisher	0.432082	1
Multi-site Trial	Visual System Disorder	Fisher	0.376331	1
Multi-site Trial	Ischemic Heart Disease	Fisher	0.530307	1
Multi-site Trial	Antithrombotic Agents	Chi-squared	0.006242	1
Multi-site Trial	Antineoplastic Agents	Chi-squared	0.126398	1
Multi-site Trial	Calcium Channel Blockers	Chi-squared	0.633781	1

© 2021 Rogers JR et al. *JAMA Network Open*.

Trial Characteristic	Covariate Label	Statistical Test	P-value	Adjusted P-value
Multi-site Trial	Agents Acting On The Renin-Angiotensin System	Chi-squared	0.869185	1
Multi-site Trial	Lipid Modifying Agents	Chi-squared	0.416677	1
Multi-site Trial	Antibacterials For Systemic Use	Chi-squared	0.009748	1
Multi-site Trial	Drugs For Obstructive Airway Diseases	Chi-squared	0.201404	1
Multi-site Trial	Antiinflammatory And Antirheumatic Products	Chi-squared	0.804199	1
Multi-site Trial	Opioids	Chi-squared	0.011306	1
Multi-site Trial	Antiepileptics	Chi-squared	0.367985	1
Multi-site Trial	Psycholeptics	Chi-squared	0.010307	1
Multi-site Trial	Antidepressants	Chi-squared	0.008851	1
Multi-site Trial	Age	Chi-squared	0.851854	1
Multi-site Trial	Ethnicity	Chi-squared	0.105647	1
Multi-site Trial	Race	Fisher	0.375447	1
Overall Enrollment	Malignant Neoplastic Disease	Chi-squared	4.33E-30	1.8E-27
Overall Enrollment	Lesion Of Liver	Chi-squared	1.6E-27	6.67E-25
Overall Enrollment	Psycholeptics	Chi-squared	7.56E-19	3.14E-16
Overall Enrollment	Drugs For Acid Related Disorders	Chi-squared	3.02E-13	1.26E-10
Overall Enrollment	Antineoplastic Agents	Chi-squared	2.12E-11	8.83E-09
Overall Enrollment	Beta Blocking Agents	Chi-squared	6.69E-11	2.78E-08
Overall Enrollment	Diuretics	Chi-squared	1.76E-09	7.33E-07
Overall Enrollment	Drugs Used In Diabetes	Chi-squared	3.72E-09	1.55E-06
Overall Enrollment	Immunosuppressants	Chi-squared	7.8E-09	3.25E-06
Overall Enrollment	Heart Disease	Chi-squared	5.18E-08	2.16E-05
Overall Enrollment	Renal Impairment	Fisher	1.19E-07	4.95E-05
Overall Enrollment	Age	Chi-squared	1.91E-07	7.94E-05
Overall Enrollment	Antidepressants	Chi-squared	4.3E-06	0.001789
Overall Enrollment	Hypertensive Disorder	Chi-squared	6.64E-06	0.00276
Overall Enrollment	Calcium Channel Blockers	Chi-squared	1.36E-05	0.005651
Overall Enrollment	Malignant Tumor Of Lung	Fisher	1.66E-05	0.006903
Overall Enrollment	Diabetes Mellitus	Chi-squared	3.69E-05	0.015337
Overall Enrollment	Gastroesophageal Reflux Disease	Fisher	9.04E-05	0.037595
Overall Enrollment	Female	Chi-squared	0.000113	0.046866
Overall Enrollment	Opioids	Chi-squared	0.00022	0.091555
Overall Enrollment	Osteoarthritis	Fisher	0.000256	0.106555
Overall Enrollment	Chronic Obstructive Lung Disease	Fisher	0.000305	0.126683
Overall Enrollment	Lipid Modifying Agents	Fisher	0.000352	0.146517
Overall Enrollment	Primary Malignant Neoplasm Of Prostate	Fisher	0.000354	0.147341
Overall Enrollment	Agents Acting On The Renin-Angiotensin System	Chi-squared	0.000476	0.197824
Overall Enrollment	Depressive Disorder	Fisher	0.000922	0.383685

© 2021 Rogers JR et al. *JAMA Network Open*.

Trial Characteristic	Covariate Label	Statistical Test	P-value	Adjusted P-value
Overall Enrollment	Race	Fisher	0.001127	0.46892
Overall Enrollment	Venous Thrombosis	Fisher	0.00172	0.715344
Overall Enrollment	Urinary Tract Infectious Disease	Fisher	0.887258	1
Overall Enrollment	Viral Hepatitis C	Chi-squared	0.077364	1
Overall Enrollment	Malignant Tumor Of Urinary Bladder	Fisher	0.634054	1
Overall Enrollment	Pneumonia	Fisher	0.04903	1
Overall Enrollment	Atrial Fibrillation	Fisher	0.559534	1
Overall Enrollment	Heart Failure	Fisher	0.010344	1
Overall Enrollment	Coronary Arteriosclerosis	Fisher	0.004042	1
Overall Enrollment	Peripheral Vascular Disease	Fisher	0.215305	1
Overall Enrollment	Cerebrovascular Disease	Fisher	0.02661	1
Overall Enrollment	Malignant Lymphoma	Fisher	0.634054	1
Overall Enrollment	Hyperlipidemia	Fisher	0.008496	1
Overall Enrollment	Obesity	Fisher	0.654221	1
Overall Enrollment	Acute Respiratory Disease	Fisher	0.023887	1
Overall Enrollment	Hematologic Neoplasm	Fisher	0.003248	1
Overall Enrollment	Malignant Tumor Of Breast	Fisher	0.342641	1
Overall Enrollment	Visual System Disorder	Fisher	0.006622	1
Overall Enrollment	Ischemic Heart Disease	Fisher	1	1
Overall Enrollment	Chronic Liver Disease	Chi-squared	0.152595	1
Overall Enrollment	Antithrombotic Agents	Chi-squared	0.009443	1
Overall Enrollment	Antibacterials For Systemic Use	Chi-squared	0.06016	1
Overall Enrollment	Drugs For Obstructive Airway Diseases	Fisher	0.207296	1
Overall Enrollment	Antiinflammatory And Antirheumatic Products	Chi-squared	0.499076	1
Overall Enrollment	Antiepileptics	Fisher	0.009792	1
Overall Enrollment	Ethnicity	Fisher	0.044372	1

© 2021 Rogers JR et al. *JAMA Network Open*.

eTable 5: Associations between trial participants' covariates and trial characteristics, inflammatory disorder trials

Trial Characteristic	Covariate Label	Statistical Test	P-value	Adjusted P-value
Phase	Viral Hepatitis C	Chi-squared	3.77E-27	1.51E-24
Phase	Chronic Liver Disease	Chi-squared	3.77E-27	1.51E-24
Phase	Lesion Of Liver	Chi-squared	2.82E-25	1.13E-22
Phase	Opioids	Chi-squared	7.26E-16	2.9E-13
Phase	Beta Blocking Agents	Chi-squared	5.6E-15	2.24E-12
Phase	Psycholeptics	Chi-squared	1.18E-14	4.73E-12
Phase	Malignant Neoplastic Disease	Chi-squared	5.88E-14	2.35E-11
Phase	Drugs For Acid Related Disorders	Chi-squared	8.43E-14	3.37E-11
Phase	Antithrombotic Agents	Chi-squared	3.05E-13	1.22E-10
Phase	Immunosuppressants	Chi-squared	4.24E-10	1.7E-07
Phase	Age	Fisher	6.65E-10	2.66E-07
Phase	Heart Disease	Chi-squared	7.75E-10	3.1E-07
Phase	Diuretics	Chi-squared	9.33E-10	3.73E-07
Phase	Drugs Used In Diabetes	Chi-squared	1.97E-08	7.86E-06
Phase	Antibacterials For Systemic Use	Chi-squared	2.05E-08	8.2E-06
Phase	Pneumonia	Chi-squared	4.71E-08	1.88E-05
Phase	Race	Fisher	9.76E-08	3.91E-05
Phase	Ethnicity	Chi-squared	1.12E-06	0.000448
Phase	Depressive Disorder	Chi-squared	1.46E-06	0.000584
Phase	Renal Impairment	Chi-squared	6.65E-05	0.026582
Phase	Female	Chi-squared	0.000118	0.047386
Phase	Calcium Channel Blockers	Chi-squared	0.000135	0.054052
Phase	Acute Respiratory Disease	Chi-squared	0.000165	0.066138
Phase	Coronary Arteriosclerosis	Fisher	0.000369	0.147602
Phase	Antidepressants	Chi-squared	0.000783	0.313369
Phase	Osteoarthritis	Chi-squared	0.040135	1
Phase	Urinary Tract Infectious Disease	Fisher	0.712912	1
Phase	Primary Malignant Neoplasm Of Prostate	Fisher	0.612319	1
Phase	Diabetes Mellitus	Chi-squared	0.010655	1
Phase	Chronic Obstructive Lung Disease	Fisher	0.021013	1
Phase	Atrial Fibrillation	Fisher	0.259322	1
Phase	Heart Failure	Fisher	0.039114	1
Phase	Hypertensive Disorder	Chi-squared	0.048394	1
Phase	Gastroesophageal Reflux Disease	Fisher	0.007567	1
Phase	Peripheral Vascular Disease	Fisher	0.193096	1
Phase	Cerebrovascular Disease	Fisher	0.003641	1
Phase	Malignant Lymphoma	Fisher	1	1

© 2021 Rogers JR et al. *JAMA Network Open*.

Trial Characteristic	Covariate Label	Statistical Test	P-value	Adjusted P-value
Phase	Hyperlipidemia	Chi-squared	0.032265	1
Phase	Obesity	Fisher	0.066432	1
Phase	Malignant Tumor Of Lung	Fisher	0.612319	1
Phase	Venous Thrombosis	Fisher	0.008808	1
Phase	Malignant Tumor Of Breast	Fisher	0.34253	1
Phase	Visual System Disorder	Fisher	0.040086	1
Phase	Ischemic Heart Disease	Fisher	0.545704	1
Phase	Antineoplastic Agents	Chi-squared	0.029125	1
Phase	Agents Acting On The Renin-Angiotensin System	Chi-squared	0.245851	1
Phase	Lipid Modifying Agents	Chi-squared	0.896583	1
Phase	Drugs For Obstructive Airway Diseases	Chi-squared	0.035196	1
Phase	Antiinflammatory And Antirheumatic Products	Chi-squared	0.50297	1
Phase	Antiepileptics	Fisher	0.172879	1
Number of Treatment Arms	Antithrombotic Agents	Chi-squared	3.66E-13	1.46E-10
Number of Treatment Arms	Opioids	Chi-squared	1.34E-12	5.37E-10
Number of Treatment Arms	Antibacterials For Systemic Use	Chi-squared	4.48E-11	1.79E-08
Number of Treatment Arms	Age	Fisher	4.19E-06	0.001675
Number of Treatment Arms	Drugs For Acid Related Disorders	Chi-squared	4.67E-06	0.001869
Number of Treatment Arms	Antineoplastic Agents	Fisher	6.86E-06	0.002743
Number of Treatment Arms	Viral Hepatitis C	Chi-squared	8.58E-06	0.003433
Number of Treatment Arms	Chronic Liver Disease	Chi-squared	8.58E-06	0.003433
Number of Treatment Arms	Immunosuppressants	Chi-squared	2.79E-05	0.011166
Number of Treatment Arms	Antiinflammatory And Antirheumatic Products	Chi-squared	0.000402	0.160661
Number of Treatment Arms	Renal Impairment	Chi-squared	0.001355	0.542078
Number of Treatment Arms	Female	Chi-squared	0.858192	1
Number of Treatment Arms	Osteoarthritis	Fisher	0.004491	1
Number of Treatment Arms	Urinary Tract Infectious Disease	Fisher	0.674375	1
Number of Treatment Arms	Primary Malignant Neoplasm Of Prostate	Fisher	1	1
Number of Treatment Arms	Diabetes Mellitus	Fisher	0.317037	1

© 2021 Rogers JR et al. *JAMA Network Open*.

Trial Characteristic	Covariate Label	Statistical Test	P-value	Adjusted P-value
Number of Treatment Arms	Chronic Obstructive Lung Disease	Fisher	0.907027	1
Number of Treatment Arms	Pneumonia	Fisher	0.002596	1
Number of Treatment Arms	Atrial Fibrillation	Fisher	0.726136	1
Number of Treatment Arms	Heart Failure	Fisher	1	1
Number of Treatment Arms	Hypertensive Disorder	Chi-squared	0.382701	1
Number of Treatment Arms	Coronary Arteriosclerosis	Fisher	0.867455	1
Number of Treatment Arms	Gastroesophageal Reflux Disease	Fisher	0.454916	1
Number of Treatment Arms	Peripheral Vascular Disease	Fisher	0.644717	1
Number of Treatment Arms	Heart Disease	Chi-squared	0.210374	1
Number of Treatment Arms	Cerebrovascular Disease	Fisher	0.223604	1
Number of Treatment Arms	Malignant Lymphoma	Fisher	1	1
Number of Treatment Arms	Hyperlipidemia	Chi-squared	0.090129	1
Number of Treatment Arms	Obesity	Fisher	0.767649	1
Number of Treatment Arms	Depressive Disorder	Fisher	0.799266	1
Number of Treatment Arms	Malignant Tumor Of Lung	Fisher	0.144928	1
Number of Treatment Arms	Malignant Neoplastic Disease	Chi-squared	0.243149	1
Number of Treatment Arms	Venous Thrombosis	Fisher	0.055789	1
Number of Treatment Arms	Acute Respiratory Disease	Fisher	0.206199	1
Number of Treatment Arms	Lesion Of Liver	Chi-squared	0.23447	1
Number of Treatment Arms	Malignant Tumor Of Breast	Fisher	0.122793	1
Number of Treatment Arms	Visual System Disorder	Fisher	0.940644	1
Number of Treatment Arms	Ischemic Heart Disease	Fisher	0.458433	1
Number of Treatment Arms	Drugs Used In Diabetes	Chi-squared	0.16993	1
Number of Treatment Arms	Diuretics	Chi-squared	0.24547	1

© 2021 Rogers JR et al. *JAMA Network Open*.

Trial Characteristic	Covariate Label	Statistical Test	P-value	Adjusted P-value
Number of Treatment Arms	Beta Blocking Agents	Chi-squared	0.167407	1
Number of Treatment Arms	Calcium Channel Blockers	Chi-squared	0.005803	1
Number of Treatment Arms	Agents Acting On The Renin-Angiotensin System	Chi-squared	0.376158	1
Number of Treatment Arms	Lipid Modifying Agents	Fisher	0.69222	1
Number of Treatment Arms	Drugs For Obstructive Airway Diseases	Chi-squared	0.075991	1
Number of Treatment Arms	Antiepileptics	Fisher	0.393674	1
Number of Treatment Arms	Psycholeptics	Chi-squared	0.161495	1
Number of Treatment Arms	Antidepressants	Chi-squared	0.871438	1
Number of Treatment Arms	Ethnicity	Chi-squared	0.991969	1
Number of Treatment Arms	Race	Fisher	0.57608	1
Randomization	Antithrombotic Agents	Chi-squared	8.98E-05	0.035937
Randomization	Female	Chi-squared	0.261759	1
Randomization	Osteoarthritis	Chi-squared	0.01059	1
Randomization	Urinary Tract Infectious Disease	Fisher	0.463561	1
Randomization	Viral Hepatitis C	Chi-squared	0.004253	1
Randomization	Primary Malignant Neoplasm Of Prostate	Fisher	0.228261	1
Randomization	Diabetes Mellitus	Chi-squared	0.418938	1
Randomization	Chronic Obstructive Lung Disease	Fisher	0.463561	1
Randomization	Pneumonia	Fisher	0.005441	1
Randomization	Atrial Fibrillation	Fisher	0.689195	1
Randomization	Heart Failure	Fisher	0.689195	1
Randomization	Hypertensive Disorder	Chi-squared	0.4865	1
Randomization	Coronary Arteriosclerosis	Fisher	0.201929	1
Randomization	Gastroesophageal Reflux Disease	Fisher	0.73885	1
Randomization	Peripheral Vascular Disease	Fisher	1	1
Randomization	Heart Disease	Chi-squared	0.022258	1
Randomization	Cerebrovascular Disease	Fisher	1	1
Randomization	Malignant Lymphoma	Fisher	1	1
Randomization	Hyperlipidemia	Chi-squared	0.01815	1
Randomization	Obesity	Fisher	1	1
Randomization	Depressive Disorder	Chi-squared	0.177934	1
Randomization	Malignant Tumor Of Lung	Fisher	0.228261	1
Randomization	Malignant Neoplastic Disease	Chi-squared	0.559217	1
Randomization	Venous Thrombosis	Fisher	1	1

© 2021 Rogers JR et al. *JAMA Network Open*.

Trial Characteristic	Covariate Label	Statistical Test	P-value	Adjusted P-value
Randomization	Acute Respiratory Disease	Chi-squared	0.181782	1
Randomization	Renal Impairment	Chi-squared	0.007087	1
Randomization	Lesion Of Liver	Chi-squared	0.048433	1
Randomization	Malignant Tumor Of Breast	Fisher	0.405059	1
Randomization	Visual System Disorder	Fisher	0.551266	1
Randomization	Ischemic Heart Disease	Fisher	0.591865	1
Randomization	Chronic Liver Disease	Chi-squared	0.004253	1
Randomization	Drugs For Acid Related Disorders	Chi-squared	0.155317	1
Randomization	Drugs Used In Diabetes	Chi-squared	0.046067	1
Randomization	Antineoplastic Agents	Chi-squared	0.12405	1
Randomization	Diuretics	Chi-squared	0.192441	1
Randomization	Beta Blocking Agents	Chi-squared	0.520521	1
Randomization	Calcium Channel Blockers	Chi-squared	0.248308	1
Randomization	Agents Acting On The Renin-Angiotensin System	Chi-squared	0.078704	1
Randomization	Lipid Modifying Agents	Chi-squared	0.074668	1
Randomization	Antibacterials For Systemic Use	Chi-squared	0.064009	1
Randomization	Drugs For Obstructive Airway Diseases	Chi-squared	0.62512	1
Randomization	Immunosuppressants	Chi-squared	0.069397	1
Randomization	Antiinflammatory And Antirheumatic Products	Chi-squared	0.538422	1
Randomization	Opioids	Chi-squared	0.130024	1
Randomization	Antiepileptics	Fisher	0.233036	1
Randomization	Psycholeptics	Chi-squared	0.803823	1
Randomization	Antidepressants	Chi-squared	1	1
Randomization	Age	Fisher	0.361012	1
Randomization	Ethnicity	Chi-squared	0.107092	1
Randomization	Race	Fisher	0.023651	1
Blinding	Heart Disease	Chi-squared	1.19E-11	4.78E-09
Blinding	Pneumonia	Chi-squared	8.22E-09	3.29E-06
Blinding	Immunosuppressants	Chi-squared	3.09E-07	0.000124
Blinding	Age	Fisher	5.69E-07	0.000228
Blinding	Lesion Of Liver	Chi-squared	6.27E-07	0.000251
Blinding	Drugs For Acid Related Disorders	Chi-squared	8.47E-07	0.000339
Blinding	Malignant Neoplastic Disease	Chi-squared	5.09E-05	0.020345
Blinding	Drugs Used In Diabetes	Chi-squared	0.000137	0.054817
Blinding	Beta Blocking Agents	Chi-squared	0.000211	0.084451
Blinding	Psycholeptics	Chi-squared	0.000629	0.251719
Blinding	Antineoplastic Agents	Chi-squared	0.001991	0.796257
Blinding	Coronary Arteriosclerosis	Fisher	0.002054	0.821635

© 2021 Rogers JR et al. *JAMA Network Open*.

Trial Characteristic	Covariate Label	Statistical Test	P-value	Adjusted P-value
Blinding	Female	Chi-squared	0.767154	1
Blinding	Osteoarthritis	Chi-squared	0.516938	1
Blinding	Urinary Tract Infectious Disease	Fisher	0.720688	1
Blinding	Viral Hepatitis C	Chi-squared	0.033924	1
Blinding	Primary Malignant Neoplasm Of Prostate	Fisher	1	1
Blinding	Diabetes Mellitus	Chi-squared	0.90819	1
Blinding	Chronic Obstructive Lung Disease	Fisher	0.131229	1
Blinding	Atrial Fibrillation	Fisher	0.051809	1
Blinding	Heart Failure	Fisher	0.010366	1
Blinding	Hypertensive Disorder	Chi-squared	0.468482	1
Blinding	Gastroesophageal Reflux Disease	Fisher	0.104114	1
Blinding	Peripheral Vascular Disease	Fisher	0.065481	1
Blinding	Cerebrovascular Disease	Fisher	0.010366	1
Blinding	Malignant Lymphoma	Fisher	0.449012	1
Blinding	Hyperlipidemia	Chi-squared	0.685158	1
Blinding	Obesity	Fisher	0.481511	1
Blinding	Depressive Disorder	Chi-squared	0.359652	1
Blinding	Malignant Tumor Of Lung	Fisher	1	1
Blinding	Venous Thrombosis	Fisher	0.051809	1
Blinding	Acute Respiratory Disease	Chi-squared	0.002983	1
Blinding	Renal Impairment	Chi-squared	0.575549	1
Blinding	Malignant Tumor Of Breast	Fisher	1	1
Blinding	Visual System Disorder	Fisher	0.153407	1
Blinding	Ischemic Heart Disease	Fisher	0.109358	1
Blinding	Chronic Liver Disease	Chi-squared	0.033924	1
Blinding	Antithrombotic Agents	Chi-squared	0.246939	1
Blinding	Diuretics	Chi-squared	0.002922	1
Blinding	Calcium Channel Blockers	Chi-squared	0.32446	1
Blinding	Agents Acting On The Renin-Angiotensin System	Chi-squared	0.13905	1
Blinding	Lipid Modifying Agents	Chi-squared	0.674626	1
Blinding	Antibacterials For Systemic Use	Chi-squared	0.019427	1
Blinding	Drugs For Obstructive Airway Diseases	Chi-squared	0.136608	1
Blinding	Antiinflammatory And Antirheumatic Products	Chi-squared	0.831736	1
Blinding	Opioids	Chi-squared	0.294229	1
Blinding	Antiepileptics	Fisher	0.775444	1
Blinding	Antidepressants	Chi-squared	0.231437	1
Blinding	Ethnicity	Chi-squared	0.008019	1
Blinding	Race	Fisher	0.004475	1

© 2021 Rogers JR et al. *JAMA Network Open*.

Trial Characteristic	Covariate Label	Statistical Test	P-value	Adjusted P-value
Industry Sponsor	Antithrombotic Agents	Chi-squared	1.92E-26	7.7E-24
Industry Sponsor	Opioids	Chi-squared	5.49E-20	2.2E-17
Industry Sponsor	Antibacterials For Systemic Use	Chi-squared	2.52E-12	1.01E-09
Industry Sponsor	Viral Hepatitis C	Chi-squared	1.33E-05	0.00534
Industry Sponsor	Chronic Liver Disease	Chi-squared	1.33E-05	0.00534
Industry Sponsor	Drugs For Acid Related Disorders	Chi-squared	2.56E-05	0.01023
Industry Sponsor	Diuretics	Chi-squared	0.000182	0.072623
Industry Sponsor	Drugs Used In Diabetes	Chi-squared	0.002162	0.865
Industry Sponsor	Female	Chi-squared	0.583828	1
Industry Sponsor	Osteoarthritis	Chi-squared	0.520804	1
Industry Sponsor	Urinary Tract Infectious Disease	Fisher	0.734101	1
Industry Sponsor	Primary Malignant Neoplasm Of Prostate	Fisher	1	1
Industry Sponsor	Diabetes Mellitus	Chi-squared	0.983815	1
Industry Sponsor	Chronic Obstructive Lung Disease	Fisher	0.305916	1
Industry Sponsor	Pneumonia	Chi-squared	0.837793	1
Industry Sponsor	Atrial Fibrillation	Fisher	0.161135	1
Industry Sponsor	Heart Failure	Fisher	0.483058	1
Industry Sponsor	Hypertensive Disorder	Chi-squared	0.211046	1
Industry Sponsor	Coronary Arteriosclerosis	Fisher	0.778858	1
Industry Sponsor	Gastroesophageal Reflux Disease	Fisher	0.555125	1
Industry Sponsor	Peripheral Vascular Disease	Fisher	0.107905	1
Industry Sponsor	Heart Disease	Chi-squared	0.030359	1
Industry Sponsor	Cerebrovascular Disease	Fisher	0.161135	1
Industry Sponsor	Malignant Lymphoma	Fisher	0.551515	1
Industry Sponsor	Hyperlipidemia	Chi-squared	0.723712	1
Industry Sponsor	Obesity	Fisher	0.755343	1
Industry Sponsor	Depressive Disorder	Chi-squared	0.389337	1
Industry Sponsor	Malignant Tumor Of Lung	Fisher	1	1
Industry Sponsor	Malignant Neoplastic Disease	Chi-squared	0.024165	1
Industry Sponsor	Venous Thrombosis	Fisher	0.006872	1
Industry Sponsor	Acute Respiratory Disease	Chi-squared	0.0447	1
Industry Sponsor	Renal Impairment	Chi-squared	0.405236	1
Industry Sponsor	Lesion Of Liver	Chi-squared	0.683169	1
Industry Sponsor	Malignant Tumor Of Breast	Fisher	1	1
Industry Sponsor	Visual System Disorder	Chi-squared	0.556172	1
Industry Sponsor	Ischemic Heart Disease	Fisher	1	1
Industry Sponsor	Antineoplastic Agents	Chi-squared	1	1
Industry Sponsor	Beta Blocking Agents	Chi-squared	0.080549	1
Industry Sponsor	Calcium Channel Blockers	Chi-squared	0.13654	1

© 2021 Rogers JR et al. *JAMA Network Open*.

Trial Characteristic	Covariate Label	Statistical Test	P-value	Adjusted P-value
Industry Sponsor	Agents Acting On The Renin-Angiotensin System	Chi-squared	0.14549	1
Industry Sponsor	Lipid Modifying Agents	Chi-squared	0.522732	1
Industry Sponsor	Drugs For Obstructive Airway Diseases	Chi-squared	0.991406	1
Industry Sponsor	Immunosuppressants	Chi-squared	0.140334	1
Industry Sponsor	Antiinflammatory And Antirheumatic Products	Chi-squared	0.374259	1
Industry Sponsor	Antiepileptics	Chi-squared	0.633634	1
Industry Sponsor	Psycholeptics	Chi-squared	0.416122	1
Industry Sponsor	Antidepressants	Chi-squared	0.485795	1
Industry Sponsor	Age	Chi-squared	0.009897	1
Industry Sponsor	Ethnicity	Chi-squared	0.222601	1
Industry Sponsor	Race	Fisher	0.258761	1
Use of DMC	Immunosuppressants	Chi-squared	5.42E-11	2.17E-08
Use of DMC	Lesion Of Liver	Chi-squared	4.91E-08	1.96E-05
Use of DMC	Malignant Neoplastic Disease	Chi-squared	4.3E-07	0.000172
Use of DMC	Viral Hepatitis C	Chi-squared	6.13E-07	0.000245
Use of DMC	Chronic Liver Disease	Chi-squared	6.13E-07	0.000245
Use of DMC	Psycholeptics	Chi-squared	4.43E-06	0.00177
Use of DMC	Drugs For Acid Related Disorders	Chi-squared	6.39E-06	0.002556
Use of DMC	Drugs Used In Diabetes	Chi-squared	8.07E-06	0.003227
Use of DMC	Beta Blocking Agents	Chi-squared	1.98E-05	0.007919
Use of DMC	Heart Disease	Chi-squared	0.000199	0.079644
Use of DMC	Opioids	Chi-squared	0.000536	0.214382
Use of DMC	Antiepileptics	Fisher	0.000619	0.247601
Use of DMC	Diuretics	Chi-squared	0.001459	0.583748
Use of DMC	Cerebrovascular Disease	Fisher	0.001471	0.588538
Use of DMC	Calcium Channel Blockers	Chi-squared	0.001769	0.707603
Use of DMC	Depressive Disorder	Chi-squared	0.001935	0.773971
Use of DMC	Race	Fisher	0.002317	0.926871
Use of DMC	Female	Chi-squared	0.21281	1
Use of DMC	Osteoarthritis	Chi-squared	0.010809	1
Use of DMC	Urinary Tract Infectious Disease	Fisher	1	1
Use of DMC	Primary Malignant Neoplasm Of Prostate	Fisher	0.26087	1
Use of DMC	Diabetes Mellitus	Chi-squared	0.356796	1
Use of DMC	Chronic Obstructive Lung Disease	Fisher	0.292956	1
Use of DMC	Pneumonia	Chi-squared	1	1
Use of DMC	Atrial Fibrillation	Fisher	0.054841	1
Use of DMC	Heart Failure	Fisher	0.246078	1
Use of DMC	Hypertensive Disorder	Chi-squared	0.125413	1

© 2021 Rogers JR et al. *JAMA Network Open*.

Trial Characteristic	Covariate Label	Statistical Test	P-value	Adjusted P-value
Use of DMC	Coronary Arteriosclerosis	Fisher	0.368297	1
Use of DMC	Gastroesophageal Reflux Disease	Fisher	0.524588	1
Use of DMC	Peripheral Vascular Disease	Fisher	0.067352	1
Use of DMC	Malignant Lymphoma	Fisher	0.454387	1
Use of DMC	Hyperlipidemia	Chi-squared	0.21805	1
Use of DMC	Obesity	Fisher	0.733606	1
Use of DMC	Malignant Tumor Of Lung	Fisher	1	1
Use of DMC	Venous Thrombosis	Fisher	0.054841	1
Use of DMC	Acute Respiratory Disease	Chi-squared	0.015971	1
Use of DMC	Renal Impairment	Chi-squared	0.983006	1
Use of DMC	Malignant Tumor Of Breast	Fisher	0.454387	1
Use of DMC	Visual System Disorder	Fisher	0.01851	1
Use of DMC	Ischemic Heart Disease	Fisher	1	1
Use of DMC	Antithrombotic Agents	Chi-squared	0.293699	1
Use of DMC	Antineoplastic Agents	Chi-squared	0.907387	1
Use of DMC	Agents Acting On The Renin-Angiotensin System	Chi-squared	0.058086	1
Use of DMC	Lipid Modifying Agents	Chi-squared	0.030897	1
Use of DMC	Antibacterials For Systemic Use	Chi-squared	0.028703	1
Use of DMC	Drugs For Obstructive Airway Diseases	Chi-squared	0.303395	1
Use of DMC	Antiinflammatory And Antirheumatic Products	Chi-squared	1	1
Use of DMC	Antidepressants	Chi-squared	0.002666	1
Use of DMC	Age	Fisher	0.003484	1
Use of DMC	Ethnicity	Chi-squared	0.007453	1
Multi-site Trial	Renal Impairment	Chi-squared	5.99E-12	2.39E-09
Multi-site Trial	Heart Disease	Chi-squared	2.53E-06	0.001013
Multi-site Trial	Diuretics	Chi-squared	4.14E-05	0.016557
Multi-site Trial	Drugs Used In Diabetes	Chi-squared	4.15E-05	0.016618
Multi-site Trial	Malignant Neoplastic Disease	Chi-squared	7.18E-05	0.028708
Multi-site Trial	Hypertensive Disorder	Chi-squared	0.000281	0.112398
Multi-site Trial	Immunosuppressants	Chi-squared	0.000531	0.21249
Multi-site Trial	Antiinflammatory And Antirheumatic Products	Chi-squared	0.001213	0.485211
Multi-site Trial	Venous Thrombosis	Fisher	0.001471	0.588538
Multi-site Trial	Lesion Of Liver	Chi-squared	0.001927	0.770652
Multi-site Trial	Female	Chi-squared	0.898445	1
Multi-site Trial	Osteoarthritis	Chi-squared	0.907387	1
Multi-site Trial	Urinary Tract Infectious Disease	Fisher	0.292956	1
Multi-site Trial	Viral Hepatitis C	Chi-squared	0.156913	1
Multi-site Trial	Primary Malignant Neoplasm Of Prostate	Fisher	1	1

© 2021 Rogers JR et al. *JAMA Network Open*.

Trial Characteristic	Covariate Label	Statistical Test	P-value	Adjusted P-value
Multi-site Trial	Diabetes Mellitus	Chi-squared	0.94805	1
Multi-site Trial	Chronic Obstructive Lung Disease	Fisher	0.13342	1
Multi-site Trial	Pneumonia	Chi-squared	1	1
Multi-site Trial	Atrial Fibrillation	Fisher	0.054841	1
Multi-site Trial	Heart Failure	Fisher	0.246078	1
Multi-site Trial	Coronary Arteriosclerosis	Fisher	0.368297	1
Multi-site Trial	Gastroesophageal Reflux Disease	Fisher	0.524588	1
Multi-site Trial	Peripheral Vascular Disease	Fisher	0.067352	1
Multi-site Trial	Cerebrovascular Disease	Fisher	0.054841	1
Multi-site Trial	Malignant Lymphoma	Fisher	0.454387	1
Multi-site Trial	Hyperlipidemia	Chi-squared	0.002629	1
Multi-site Trial	Obesity	Fisher	1	1
Multi-site Trial	Depressive Disorder	Chi-squared	0.976742	1
Multi-site Trial	Malignant Tumor Of Lung	Fisher	1	1
Multi-site Trial	Acute Respiratory Disease	Chi-squared	0.003549	1
Multi-site Trial	Malignant Tumor Of Breast	Fisher	1	1
Multi-site Trial	Visual System Disorder	Fisher	0.777312	1
Multi-site Trial	Ischemic Heart Disease	Fisher	0.608031	1
Multi-site Trial	Chronic Liver Disease	Chi-squared	0.156913	1
Multi-site Trial	Drugs For Acid Related Disorders	Chi-squared	0.09431	1
Multi-site Trial	Antithrombotic Agents	Chi-squared	0.293699	1
Multi-site Trial	Antineoplastic Agents	Chi-squared	1	1
Multi-site Trial	Beta Blocking Agents	Chi-squared	0.003069	1
Multi-site Trial	Calcium Channel Blockers	Chi-squared	0.58113	1
Multi-site Trial	Agents Acting On The Renin-Angiotensin System	Chi-squared	1	1
Multi-site Trial	Lipid Modifying Agents	Chi-squared	0.424251	1
Multi-site Trial	Antibacterials For Systemic Use	Chi-squared	0.995244	1
Multi-site Trial	Drugs For Obstructive Airway Diseases	Chi-squared	1	1
Multi-site Trial	Opioids	Chi-squared	0.513535	1
Multi-site Trial	Antiepileptics	Fisher	0.396132	1
Multi-site Trial	Psycholeptics	Chi-squared	0.039617	1
Multi-site Trial	Antidepressants	Chi-squared	0.132066	1
Multi-site Trial	Age	Fisher	0.029198	1
Multi-site Trial	Ethnicity	Chi-squared	0.618841	1
Multi-site Trial	Race	Fisher	0.914445	1
Overall Enrollment	Renal Impairment	Chi-squared	6.71E-26	2.68E-23
Overall Enrollment	Lesion Of Liver	Chi-squared	7.92E-19	3.17E-16
Overall Enrollment	Immunosuppressants	Chi-squared	1.96E-15	7.83E-13
Overall Enrollment	Diuretics	Chi-squared	2.75E-11	1.1E-08

© 2021 Rogers JR et al. *JAMA Network Open*.

Trial Characteristic	Covariate Label	Statistical Test	P-value	Adjusted P-value
Overall Enrollment	Viral Hepatitis C	Chi-squared	1.15E-10	4.59E-08
Overall Enrollment	Chronic Liver Disease	Chi-squared	1.15E-10	4.59E-08
Overall Enrollment	Beta Blocking Agents	Chi-squared	3.8E-09	1.52E-06
Overall Enrollment	Hyperlipidemia	Chi-squared	8.44E-09	3.38E-06
Overall Enrollment	Malignant Neoplastic Disease	Chi-squared	2.56E-08	1.02E-05
Overall Enrollment	Drugs Used In Diabetes	Chi-squared	5.15E-07	0.000206
Overall Enrollment	Antibacterials For Systemic Use	Chi-squared	1.14E-05	0.004568
Overall Enrollment	Psycholeptics	Chi-squared	5.15E-05	0.0206
Overall Enrollment	Drugs For Acid Related Disorders	Chi-squared	0.000139	0.05563
Overall Enrollment	Age	Fisher	0.000179	0.071721
Overall Enrollment	Acute Respiratory Disease	Fisher	0.00032	0.128171
Overall Enrollment	Antineoplastic Agents	Fisher	0.000488	0.195028
Overall Enrollment	Hypertensive Disorder	Chi-squared	0.000603	0.241208
Overall Enrollment	Agents Acting On The Renin-Angiotensin System	Chi-squared	0.000819	0.327654
Overall Enrollment	Heart Disease	Chi-squared	0.000822	0.328923
Overall Enrollment	Diabetes Mellitus	Fisher	0.002419	0.967688
Overall Enrollment	Female	Chi-squared	0.057205	1
Overall Enrollment	Osteoarthritis	Fisher	0.087682	1
Overall Enrollment	Urinary Tract Infectious Disease	Fisher	0.185994	1
Overall Enrollment	Primary Malignant Neoplasm Of Prostate	Fisher	1	1
Overall Enrollment	Chronic Obstructive Lung Disease	Fisher	0.00985	1
Overall Enrollment	Pneumonia	Fisher	0.181808	1
Overall Enrollment	Atrial Fibrillation	Fisher	0.456996	1
Overall Enrollment	Heart Failure	Fisher	0.130946	1
Overall Enrollment	Coronary Arteriosclerosis	Fisher	0.211083	1
Overall Enrollment	Gastroesophageal Reflux Disease	Fisher	0.784352	1
Overall Enrollment	Peripheral Vascular Disease	Fisher	0.62029	1
Overall Enrollment	Cerebrovascular Disease	Fisher	0.260328	1
Overall Enrollment	Malignant Lymphoma	Fisher	0.62029	1
Overall Enrollment	Obesity	Fisher	1	1
Overall Enrollment	Depressive Disorder	Fisher	0.021486	1
Overall Enrollment	Malignant Tumor Of Lung	Fisher	0.126812	1
Overall Enrollment	Venous Thrombosis	Fisher	0.01009	1
Overall Enrollment	Malignant Tumor Of Breast	Fisher	1	1
Overall Enrollment	Visual System Disorder	Fisher	0.002858	1
Overall Enrollment	Ischemic Heart Disease	Fisher	0.686629	1
Overall Enrollment	Antithrombotic Agents	Chi-squared	0.313273	1
Overall Enrollment	Calcium Channel Blockers	Chi-squared	0.004258	1
Overall Enrollment	Lipid Modifying Agents	Fisher	0.003564	1

© 2021 Rogers JR et al. *JAMA Network Open*.

Trial Characteristic	Covariate Label	Statistical Test	P-value	Adjusted P-value
Overall Enrollment	Drugs For Obstructive Airway Diseases	Fisher	0.506001	1
Overall Enrollment	Antiinflammatory And Antirheumatic Products	Chi-squared	0.038167	1
Overall Enrollment	Opioids	Chi-squared	0.209308	1
Overall Enrollment	Antiepileptics	Fisher	0.645338	1
Overall Enrollment	Antidepressants	Chi-squared	0.021528	1
Overall Enrollment	Ethnicity	Chi-squared	0.004378	1
Overall Enrollment	Race	Fisher	0.003717	1

© 2021 Rogers JR et al. *JAMA Network Open*.

eTable 6: Associations between trial participants' covariates and trial characteristics, disorder of cardiovascular system trials

Trial Characteristic	Covariate Label	Statistical Test	P-value	Adjusted P-value
Phase	Peripheral Vascular Disease	Fisher	2.8E-26	1.14E-23
Phase	Hypertensive Disorder	Fisher	4.26E-19	1.74E-16
Phase	Heart Failure	Fisher	1.71E-15	6.98E-13
Phase	Hyperlipidemia	Fisher	2.53E-10	1.03E-07
Phase	Coronary Arteriosclerosis	Fisher	1.24E-08	5.04E-06
Phase	Heart Disease	Fisher	5.09E-08	2.08E-05
Phase	Calcium Channel Blockers	Fisher	6.91E-08	2.82E-05
Phase	Pneumonia	Fisher	0.000405	0.165437
Phase	Venous Thrombosis	Fisher	0.000483	0.196924
Phase	Antiepileptics	Fisher	0.000683	0.278525
Phase	Diuretics	Fisher	0.001288	0.52539
Phase	Antiinflammatory And Antirheumatic Products	Fisher	0.002449	0.999158
Phase	Female	Fisher	0.008849	1
Phase	Osteoarthritis	Fisher	0.653712	1
Phase	Urinary Tract Infectious Disease	Fisher	0.840347	1
Phase	Viral Hepatitis C	Fisher	0.224727	1
Phase	Malignant Tumor Of Urinary Bladder	Fisher	1	1
Phase	Primary Malignant Neoplasm Of Prostate	Fisher	0.448225	1
Phase	Diabetes Mellitus	Fisher	0.261214	1
Phase	Chronic Obstructive Lung Disease	Fisher	0.006547	1
Phase	Atrial Fibrillation	Fisher	0.050167	1
Phase	Gastroesophageal Reflux Disease	Fisher	0.722258	1
Phase	Cerebrovascular Disease	Fisher	0.608063	1
Phase	Malignant Lymphoma	Fisher	0.125214	1
Phase	Obesity	Fisher	0.251536	1
Phase	Depressive Disorder	Fisher	0.28784	1
Phase	Malignant Neoplastic Disease	Fisher	0.881352	1
Phase	Acute Respiratory Disease	Fisher	0.121807	1
Phase	Renal Impairment	Fisher	0.027641	1
Phase	Hematologic Neoplasm	Fisher	0.69239	1
Phase	Lesion Of Liver	Fisher	0.495298	1
Phase	Malignant Tumor Of Breast	Fisher	0.69239	1
Phase	Visual System Disorder	Fisher	0.009518	1
Phase	Ischemic Heart Disease	Fisher	0.008159	1
Phase	Chronic Liver Disease	Fisher	0.006218	1
Phase	Drugs For Acid Related Disorders	Fisher	0.516718	1
Phase	Drugs Used In Diabetes	Fisher	0.097721	1

© 2021 Rogers JR et al. *JAMA Network Open*.

Trial Characteristic	Covariate Label	Statistical Test	P-value	Adjusted P-value
Phase	Antithrombotic Agents	Fisher	0.383853	1
Phase	Antineoplastic Agents	Fisher	0.497762	1
Phase	Beta Blocking Agents	Fisher	0.799588	1
Phase	Agents Acting On The Renin-Angiotensin System	Fisher	0.007769	1
Phase	Lipid Modifying Agents	Fisher	0.089593	1
Phase	Antibacterials For Systemic Use	Fisher	0.1126	1
Phase	Drugs For Obstructive Airway Diseases	Fisher	0.173558	1
Phase	Immunosuppressants	Fisher	0.283312	1
Phase	Opioids	Fisher	0.159449	1
Phase	Psycholeptics	Fisher	0.042932	1
Phase	Antidepressants	Fisher	0.352	1
Phase	Age	Fisher	0.014582	1
Phase	Ethnicity	Fisher	0.052773	1
Phase	Race	Fisher	0.034803	1
Number of Treatment Arms	Atrial Fibrillation	Chi-squared	1.19E-13	4.84E-11
Number of Treatment Arms	Age	Fisher	3.14E-13	1.28E-10
Number of Treatment Arms	Hypertensive Disorder	Chi-squared	8.09E-10	3.3E-07
Number of Treatment Arms	Peripheral Vascular Disease	Chi-squared	2.67E-09	1.09E-06
Number of Treatment Arms	Heart Failure	Chi-squared	1.44E-08	5.87E-06
Number of Treatment Arms	Diuretics	Chi-squared	3.72E-07	0.000152
Number of Treatment Arms	Heart Disease	Fisher	6.21E-07	0.000253
Number of Treatment Arms	Female	Chi-squared	9.53E-07	0.000389
Number of Treatment Arms	Hyperlipidemia	Chi-squared	2.16E-05	0.008809
Number of Treatment Arms	Agents Acting On The Renin-Angiotensin System	Chi-squared	0.00012	0.048944
Number of Treatment Arms	Drugs Used In Diabetes	Chi-squared	0.000126	0.051482
Number of Treatment Arms	Diabetes Mellitus	Chi-squared	0.000229	0.093508
Number of Treatment Arms	Ethnicity	Fisher	0.000665	0.27117
Number of Treatment Arms	Ischemic Heart Disease	Chi-squared	0.000835	0.34076
Number of Treatment Arms	Osteoarthritis	Fisher	0.153406	1

© 2021 Rogers JR et al. *JAMA Network Open*.

Trial Characteristic	Covariate Label	Statistical Test	P-value	Adjusted P-value
Number of Treatment Arms	Urinary Tract Infectious Disease	Fisher	0.037511	1
Number of Treatment Arms	Viral Hepatitis C	Fisher	0.648051	1
Number of Treatment Arms	Malignant Tumor Of Urinary Bladder	Fisher	1	1
Number of Treatment Arms	Primary Malignant Neoplasm Of Prostate	Fisher	0.23319	1
Number of Treatment Arms	Chronic Obstructive Lung Disease	Fisher	0.461532	1
Number of Treatment Arms	Pneumonia	Fisher	0.457101	1
Number of Treatment Arms	Coronary Arteriosclerosis	Chi-squared	0.307221	1
Number of Treatment Arms	Gastroesophageal Reflux Disease	Fisher	0.359147	1
Number of Treatment Arms	Cerebrovascular Disease	Fisher	0.140202	1
Number of Treatment Arms	Malignant Lymphoma	Fisher	0.234811	1
Number of Treatment Arms	Obesity	Fisher	0.010064	1
Number of Treatment Arms	Depressive Disorder	Fisher	0.023536	1
Number of Treatment Arms	Malignant Neoplastic Disease	Fisher	0.76089	1
Number of Treatment Arms	Venous Thrombosis	Fisher	1	1
Number of Treatment Arms	Acute Respiratory Disease	Fisher	0.865903	1
Number of Treatment Arms	Renal Impairment	Fisher	0.180482	1
Number of Treatment Arms	Hematologic Neoplasm	Fisher	1	1
Number of Treatment Arms	Lesion Of Liver	Fisher	1	1
Number of Treatment Arms	Malignant Tumor Of Breast	Fisher	0.648051	1
Number of Treatment Arms	Visual System Disorder	Fisher	0.284619	1
Number of Treatment Arms	Chronic Liver Disease	Fisher	0.336585	1
Number of Treatment Arms	Drugs For Acid Related Disorders	Chi-squared	0.107362	1
Number of Treatment Arms	Antithrombotic Agents	Fisher	0.238767	1
Number of Treatment Arms	Antineoplastic Agents	Fisher	0.032073	1

© 2021 Rogers JR et al. *JAMA Network Open*.

Trial Characteristic	Covariate Label	Statistical Test	P-value	Adjusted P-value
Number of Treatment Arms	Beta Blocking Agents	Chi-squared	0.062731	1
Number of Treatment Arms	Calcium Channel Blockers	Chi-squared	0.108154	1
Number of Treatment Arms	Lipid Modifying Agents	Chi-squared	0.090475	1
Number of Treatment Arms	Antibacterials For Systemic Use	Chi-squared	0.967152	1
Number of Treatment Arms	Drugs For Obstructive Airway Diseases	Chi-squared	0.252013	1
Number of Treatment Arms	Immunosuppressants	Fisher	0.304646	1
Number of Treatment Arms	Antiinflammatory And Antirheumatic Products	Chi-squared	0.588699	1
Number of Treatment Arms	Opioids	Chi-squared	0.006165	1
Number of Treatment Arms	Antiepileptics	Fisher	0.46327	1
Number of Treatment Arms	Psycholeptics	Chi-squared	0.887236	1
Number of Treatment Arms	Antidepressants	Fisher	0.421852	1
Number of Treatment Arms	Race	Fisher	0.013702	1
Randomization	Age	Fisher	9.68E-13	3.95E-10
Randomization	Atrial Fibrillation	Chi-squared	1.03E-12	4.2E-10
Randomization	Hypertensive Disorder	Chi-squared	1.44E-08	5.88E-06
Randomization	Peripheral Vascular Disease	Chi-squared	2.07E-07	8.46E-05
Randomization	Hyperlipidemia	Chi-squared	7.7E-06	0.003143
Randomization	Heart Failure	Chi-squared	2.1E-05	0.008576
Randomization	Female	Chi-squared	2.46E-05	0.010056
Randomization	Agents Acting On The Renin-Angiotensin System	Chi-squared	4.71E-05	0.019222
Randomization	Drugs Used In Diabetes	Chi-squared	5.28E-05	0.021542
Randomization	Heart Disease	Chi-squared	0.000148	0.060502
Randomization	Ischemic Heart Disease	Chi-squared	0.000334	0.136138
Randomization	Diabetes Mellitus	Chi-squared	0.000605	0.246723
Randomization	Diuretics	Chi-squared	0.000683	0.278764
Randomization	Ethnicity	Chi-squared	0.001316	0.537043
Randomization	Osteoarthritis	Chi-squared	1	1
Randomization	Urinary Tract Infectious Disease	Fisher	0.037706	1
Randomization	Viral Hepatitis C	Fisher	0.559682	1
Randomization	Malignant Tumor Of Urinary Bladder	Fisher	1	1
Randomization	Primary Malignant Neoplasm Of Prostate	Fisher	0.559682	1
Randomization	Chronic Obstructive Lung Disease	Chi-squared	0.309911	1

© 2021 Rogers JR et al. *JAMA Network Open*.

Trial Characteristic	Covariate Label	Statistical Test	P-value	Adjusted P-value
Randomization	Pneumonia	Chi-squared	1	1
Randomization	Coronary Arteriosclerosis	Chi-squared	0.169261	1
Randomization	Gastroesophageal Reflux Disease	Chi-squared	0.260212	1
Randomization	Cerebrovascular Disease	Chi-squared	0.133191	1
Randomization	Malignant Lymphoma	Fisher	0.628424	1
Randomization	Obesity	Chi-squared	0.021579	1
Randomization	Depressive Disorder	Chi-squared	0.070898	1
Randomization	Malignant Neoplastic Disease	Chi-squared	0.78154	1
Randomization	Venous Thrombosis	Fisher	1	1
Randomization	Acute Respiratory Disease	Chi-squared	0.70901	1
Randomization	Renal Impairment	Chi-squared	0.804435	1
Randomization	Hematologic Neoplasm	Fisher	1	1
Randomization	Lesion Of Liver	Fisher	1	1
Randomization	Malignant Tumor Of Breast	Fisher	0.559682	1
Randomization	Visual System Disorder	Chi-squared	0.185518	1
Randomization	Chronic Liver Disease	Fisher	1	1
Randomization	Drugs For Acid Related Disorders	Chi-squared	0.687608	1
Randomization	Antithrombotic Agents	Chi-squared	0.686691	1
Randomization	Antineoplastic Agents	Fisher	0.326518	1
Randomization	Beta Blocking Agents	Chi-squared	0.033754	1
Randomization	Calcium Channel Blockers	Chi-squared	0.272044	1
Randomization	Lipid Modifying Agents	Chi-squared	0.186938	1
Randomization	Antibacterials For Systemic Use	Chi-squared	0.895895	1
Randomization	Drugs For Obstructive Airway Diseases	Chi-squared	0.230792	1
Randomization	Immunosuppressants	Fisher	0.291436	1
Randomization	Antiinflammatory And Antirheumatic Products	Chi-squared	0.391713	1
Randomization	Opioids	Chi-squared	0.008908	1
Randomization	Antiepileptics	Chi-squared	0.320865	1
Randomization	Psycholeptics	Chi-squared	0.930369	1
Randomization	Antidepressants	Chi-squared	0.247805	1
Randomization	Race	Fisher	0.205448	1
Blinding	Hyperlipidemia	Chi-squared	3.34E-12	1.36E-09
Blinding	Peripheral Vascular Disease	Chi-squared	7.18E-12	2.93E-09
Blinding	Atrial Fibrillation	Chi-squared	9.39E-10	3.83E-07
Blinding	Agents Acting On The Renin-Angiotensin System	Chi-squared	1.11E-09	4.52E-07
Blinding	Drugs Used In Diabetes	Chi-squared	2.54E-09	1.04E-06
Blinding	Diabetes Mellitus	Chi-squared	2.32E-07	9.46E-05
Blinding	Hypertensive Disorder	Chi-squared	4.35E-07	0.000177

© 2021 Rogers JR et al. *JAMA Network Open*.

Trial Characteristic	Covariate Label	Statistical Test	P-value	Adjusted P-value
Blinding	Lipid Modifying Agents	Chi-squared	1.28E-06	0.000523
Blinding	Ischemic Heart Disease	Chi-squared	1.64E-06	0.000669
Blinding	Coronary Arteriosclerosis	Chi-squared	0.001138	0.464233
Blinding	Age	Fisher	0.001703	0.694643
Blinding	Female	Chi-squared	0.103951	1
Blinding	Osteoarthritis	Chi-squared	0.733793	1
Blinding	Urinary Tract Infectious Disease	Fisher	0.752423	1
Blinding	Viral Hepatitis C	Fisher	0.29615	1
Blinding	Malignant Tumor Of Urinary Bladder	Fisher	1	1
Blinding	Primary Malignant Neoplasm Of Prostate	Fisher	0.29615	1
Blinding	Chronic Obstructive Lung Disease	Chi-squared	0.057212	1
Blinding	Pneumonia	Chi-squared	0.119508	1
Blinding	Heart Failure	Chi-squared	0.217718	1
Blinding	Gastroesophageal Reflux Disease	Chi-squared	0.071286	1
Blinding	Heart Disease	Chi-squared	0.869485	1
Blinding	Cerebrovascular Disease	Chi-squared	0.098991	1
Blinding	Malignant Lymphoma	Fisher	1	1
Blinding	Obesity	Chi-squared	0.283779	1
Blinding	Depressive Disorder	Chi-squared	0.027814	1
Blinding	Malignant Neoplastic Disease	Chi-squared	0.275904	1
Blinding	Venous Thrombosis	Fisher	0.006767	1
Blinding	Acute Respiratory Disease	Chi-squared	0.619686	1
Blinding	Renal Impairment	Chi-squared	0.96735	1
Blinding	Hematologic Neoplasm	Fisher	1	1
Blinding	Lesion Of Liver	Fisher	1	1
Blinding	Malignant Tumor Of Breast	Fisher	0.29615	1
Blinding	Visual System Disorder	Chi-squared	0.051813	1
Blinding	Chronic Liver Disease	Fisher	1	1
Blinding	Drugs For Acid Related Disorders	Chi-squared	0.921587	1
Blinding	Antithrombotic Agents	Chi-squared	0.016217	1
Blinding	Antineoplastic Agents	Fisher	0.161445	1
Blinding	Diuretics	Chi-squared	1	1
Blinding	Beta Blocking Agents	Chi-squared	0.009322	1
Blinding	Calcium Channel Blockers	Chi-squared	1	1
Blinding	Antibacterials For Systemic Use	Chi-squared	0.498488	1
Blinding	Drugs For Obstructive Airway Diseases	Chi-squared	0.012427	1
Blinding	Immunosuppressants	Fisher	0.331654	1
Blinding	Antiinflammatory And Antirheumatic Products	Chi-squared	0.671162	1
Blinding	Opioids	Chi-squared	0.128446	1

© 2021 Rogers JR et al. *JAMA Network Open*.

Trial Characteristic	Covariate Label	Statistical Test	P-value	Adjusted P-value
Blinding	Antiepileptics	Chi-squared	1	1
Blinding	Psycholeptics	Chi-squared	0.96083	1
Blinding	Antidepressants	Chi-squared	0.038049	1
Blinding	Ethnicity	Chi-squared	0.007666	1
Blinding	Race	Chi-squared	0.054974	1
Industry Sponsor	Heart Disease	Fisher	4.72E-16	1.93E-13
Industry Sponsor	Female	Chi-squared	1.05E-09	4.29E-07
Industry Sponsor	Antithrombotic Agents	Chi-squared	1.06E-09	4.32E-07
Industry Sponsor	Lipid Modifying Agents	Chi-squared	9.78E-08	3.99E-05
Industry Sponsor	Hypertensive Disorder	Chi-squared	1.05E-05	0.004264
Industry Sponsor	Drugs Used In Diabetes	Chi-squared	1.05E-05	0.004264
Industry Sponsor	Heart Failure	Chi-squared	4.43E-05	0.018085
Industry Sponsor	Obesity	Fisher	0.0002	0.081708
Industry Sponsor	Coronary Arteriosclerosis	Chi-squared	0.000214	0.087297
Industry Sponsor	Ischemic Heart Disease	Chi-squared	0.00026	0.105991
Industry Sponsor	Diabetes Mellitus	Chi-squared	0.000302	0.12316
Industry Sponsor	Peripheral Vascular Disease	Chi-squared	0.000329	0.134381
Industry Sponsor	Atrial Fibrillation	Chi-squared	0.000855	0.348664
Industry Sponsor	Osteoarthritis	Fisher	0.069886	1
Industry Sponsor	Urinary Tract Infectious Disease	Fisher	0.303972	1
Industry Sponsor	Viral Hepatitis C	Fisher	0.027486	1
Industry Sponsor	Malignant Tumor Of Urinary Bladder	Fisher	1	1
Industry Sponsor	Primary Malignant Neoplasm Of Prostate	Fisher	1	1
Industry Sponsor	Chronic Obstructive Lung Disease	Fisher	0.742532	1
Industry Sponsor	Pneumonia	Fisher	0.149413	1
Industry Sponsor	Gastroesophageal Reflux Disease	Fisher	0.149959	1
Industry Sponsor	Cerebrovascular Disease	Fisher	0.099412	1
Industry Sponsor	Malignant Lymphoma	Fisher	1	1
Industry Sponsor	Hyperlipidemia	Chi-squared	0.255043	1
Industry Sponsor	Depressive Disorder	Fisher	0.73441	1
Industry Sponsor	Malignant Neoplastic Disease	Fisher	1	1
Industry Sponsor	Venous Thrombosis	Fisher	0.606414	1
Industry Sponsor	Acute Respiratory Disease	Fisher	0.240216	1
Industry Sponsor	Renal Impairment	Chi-squared	0.877808	1
Industry Sponsor	Hematologic Neoplasm	Fisher	1	1
Industry Sponsor	Lesion Of Liver	Fisher	1	1
Industry Sponsor	Malignant Tumor Of Breast	Fisher	0.272529	1
Industry Sponsor	Visual System Disorder	Fisher	0.061475	1
Industry Sponsor	Chronic Liver Disease	Fisher	0.346197	1
Industry Sponsor	Drugs For Acid Related Disorders	Chi-squared	0.151147	1

© 2021 Rogers JR et al. *JAMA Network Open*.

Trial Characteristic	Covariate Label	Statistical Test	P-value	Adjusted P-value
Industry Sponsor	Antineoplastic Agents	Fisher	1	1
Industry Sponsor	Diuretics	Chi-squared	0.065462	1
Industry Sponsor	Beta Blocking Agents	Chi-squared	0.014438	1
Industry Sponsor	Calcium Channel Blockers	Chi-squared	0.54739	1
Industry Sponsor	Agents Acting On The Renin-Angiotensin System	Chi-squared	0.006104	1
Industry Sponsor	Antibacterials For Systemic Use	Chi-squared	0.19791	1
Industry Sponsor	Drugs For Obstructive Airway Diseases	Chi-squared	0.189081	1
Industry Sponsor	Immunosuppressants	Fisher	0.606414	1
Industry Sponsor	Antiinflammatory And Antirheumatic Products	Chi-squared	0.00819	1
Industry Sponsor	Opioids	Chi-squared	0.178448	1
Industry Sponsor	Antiepileptics	Chi-squared	0.071504	1
Industry Sponsor	Psycholeptics	Chi-squared	0.245969	1
Industry Sponsor	Antidepressants	Fisher	0.442088	1
Industry Sponsor	Age	Fisher	0.043163	1
Industry Sponsor	Ethnicity	Chi-squared	0.450677	1
Industry Sponsor	Race	Fisher	0.002518	1
Use of DMC	Heart Failure	Chi-squared	3.38E-08	1.38E-05
Use of DMC	Calcium Channel Blockers	Chi-squared	0.000115	0.047056
Use of DMC	Cerebrovascular Disease	Chi-squared	0.000274	0.111959
Use of DMC	Age	Fisher	0.00123	0.501694
Use of DMC	Female	Chi-squared	0.896932	1
Use of DMC	Osteoarthritis	Fisher	0.40884	1
Use of DMC	Urinary Tract Infectious Disease	Fisher	0.030669	1
Use of DMC	Viral Hepatitis C	Fisher	1	1
Use of DMC	Malignant Tumor Of Urinary Bladder	Fisher	0.119122	1
Use of DMC	Primary Malignant Neoplasm Of Prostate	Fisher	1	1
Use of DMC	Diabetes Mellitus	Chi-squared	0.435113	1
Use of DMC	Chronic Obstructive Lung Disease	Fisher	1	1
Use of DMC	Pneumonia	Fisher	0.507209	1
Use of DMC	Atrial Fibrillation	Chi-squared	1	1
Use of DMC	Hypertensive Disorder	Chi-squared	0.008851	1
Use of DMC	Coronary Arteriosclerosis	Chi-squared	0.004817	1
Use of DMC	Gastroesophageal Reflux Disease	Fisher	0.783675	1
Use of DMC	Peripheral Vascular Disease	Chi-squared	0.004428	1
Use of DMC	Heart Disease	Fisher	0.012642	1
Use of DMC	Malignant Lymphoma	Fisher	0.109661	1
Use of DMC	Hyperlipidemia	Chi-squared	0.013327	1
Use of DMC	Obesity	Fisher	0.40884	1

© 2021 Rogers JR et al. *JAMA Network Open*.

Trial Characteristic	Covariate Label	Statistical Test	P-value	Adjusted P-value
Use of DMC	Depressive Disorder	Fisher	0.104142	1
Use of DMC	Malignant Neoplastic Disease	Fisher	0.088825	1
Use of DMC	Venous Thrombosis	Fisher	0.614605	1
Use of DMC	Acute Respiratory Disease	Fisher	0.487315	1
Use of DMC	Renal Impairment	Chi-squared	0.854541	1
Use of DMC	Hematologic Neoplasm	Fisher	1	1
Use of DMC	Lesion Of Liver	Fisher	1	1
Use of DMC	Malignant Tumor Of Breast	Fisher	0.31736	1
Use of DMC	Visual System Disorder	Fisher	0.780316	1
Use of DMC	Ischemic Heart Disease	Chi-squared	0.970568	1
Use of DMC	Chronic Liver Disease	Fisher	1	1
Use of DMC	Drugs For Acid Related Disorders	Chi-squared	0.345671	1
Use of DMC	Drugs Used In Diabetes	Chi-squared	0.549281	1
Use of DMC	Antithrombotic Agents	Chi-squared	0.309939	1
Use of DMC	Antineoplastic Agents	Fisher	1	1
Use of DMC	Diuretics	Chi-squared	0.217653	1
Use of DMC	Beta Blocking Agents	Chi-squared	0.30347	1
Use of DMC	Agents Acting On The Renin-Angiotensin System	Chi-squared	1	1
Use of DMC	Lipid Modifying Agents	Chi-squared	0.044421	1
Use of DMC	Antibacterials For Systemic Use	Chi-squared	0.140998	1
Use of DMC	Drugs For Obstructive Airway Diseases	Chi-squared	0.496822	1
Use of DMC	Immunosuppressants	Fisher	0.614605	1
Use of DMC	Antiinflammatory And Antirheumatic Products	Chi-squared	0.020028	1
Use of DMC	Opioids	Chi-squared	0.756962	1
Use of DMC	Antiepileptics	Chi-squared	0.100359	1
Use of DMC	Psycholeptics	Chi-squared	0.825899	1
Use of DMC	Antidepressants	Chi-squared	0.178634	1
Use of DMC	Ethnicity	Chi-squared	0.003251	1
Use of DMC	Race	Fisher	0.047092	1
Multi-site Trial	Coronary Arteriosclerosis	Chi-squared	7.75E-05	0.031605
Multi-site Trial	Antithrombotic Agents	Chi-squared	8.21E-05	0.033479
Multi-site Trial	Peripheral Vascular Disease	Chi-squared	0.000117	0.047855
Multi-site Trial	Age	Fisher	0.002245	0.915934
Multi-site Trial	Female	Chi-squared	0.003657	1
Multi-site Trial	Osteoarthritis	Chi-squared	0.01925	1
Multi-site Trial	Urinary Tract Infectious Disease	Fisher	0.029908	1
Multi-site Trial	Viral Hepatitis C	Fisher	0.08348	1
Multi-site Trial	Malignant Tumor Of Urinary Bladder	Fisher	0.178683	1

© 2021 Rogers JR et al. *JAMA Network Open*.

Trial Characteristic	Covariate Label	Statistical Test	P-value	Adjusted P-value
Multi-site Trial	Primary Malignant Neoplasm Of Prostate	Fisher	0.447111	1
Multi-site Trial	Diabetes Mellitus	Chi-squared	0.091399	1
Multi-site Trial	Chronic Obstructive Lung Disease	Fisher	1	1
Multi-site Trial	Pneumonia	Fisher	1	1
Multi-site Trial	Atrial Fibrillation	Chi-squared	0.005014	1
Multi-site Trial	Heart Failure	Chi-squared	0.007165	1
Multi-site Trial	Hypertensive Disorder	Chi-squared	0.81756	1
Multi-site Trial	Gastroesophageal Reflux Disease	Chi-squared	0.372028	1
Multi-site Trial	Heart Disease	Chi-squared	0.293561	1
Multi-site Trial	Cerebrovascular Disease	Chi-squared	0.049327	1
Multi-site Trial	Malignant Lymphoma	Fisher	1	1
Multi-site Trial	Hyperlipidemia	Chi-squared	0.561971	1
Multi-site Trial	Obesity	Chi-squared	0.060278	1
Multi-site Trial	Depressive Disorder	Fisher	0.030252	1
Multi-site Trial	Malignant Neoplastic Disease	Fisher	0.553193	1
Multi-site Trial	Venous Thrombosis	Fisher	0.218482	1
Multi-site Trial	Acute Respiratory Disease	Fisher	0.546434	1
Multi-site Trial	Renal Impairment	Chi-squared	1	1
Multi-site Trial	Hematologic Neoplasm	Fisher	1	1
Multi-site Trial	Lesion Of Liver	Fisher	1	1
Multi-site Trial	Malignant Tumor Of Breast	Fisher	0.447111	1
Multi-site Trial	Visual System Disorder	Chi-squared	0.104791	1
Multi-site Trial	Ischemic Heart Disease	Chi-squared	0.570918	1
Multi-site Trial	Chronic Liver Disease	Fisher	0.546841	1
Multi-site Trial	Drugs For Acid Related Disorders	Chi-squared	0.087625	1
Multi-site Trial	Drugs Used In Diabetes	Chi-squared	0.081555	1
Multi-site Trial	Antineoplastic Agents	Fisher	0.589999	1
Multi-site Trial	Diuretics	Chi-squared	0.181486	1
Multi-site Trial	Beta Blocking Agents	Chi-squared	0.648779	1
Multi-site Trial	Calcium Channel Blockers	Chi-squared	0.012155	1
Multi-site Trial	Agents Acting On The Renin-Angiotensin System	Chi-squared	1	1
Multi-site Trial	Lipid Modifying Agents	Chi-squared	0.005425	1
Multi-site Trial	Antibacterials For Systemic Use	Chi-squared	0.133361	1
Multi-site Trial	Drugs For Obstructive Airway Diseases	Chi-squared	0.345068	1
Multi-site Trial	Immunosuppressants	Fisher	0.218482	1
Multi-site Trial	Antiinflammatory And Antirheumatic Products	Chi-squared	0.212875	1
Multi-site Trial	Opioids	Chi-squared	0.20384	1
Multi-site Trial	Antiepileptics	Chi-squared	0.002802	1

© 2021 Rogers JR et al. *JAMA Network Open*.

Trial Characteristic	Covariate Label	Statistical Test	P-value	Adjusted P-value
Multi-site Trial	Psycholeptics	Chi-squared	0.316067	1
Multi-site Trial	Antidepressants	Chi-squared	0.232105	1
Multi-site Trial	Ethnicity	Chi-squared	0.20867	1
Multi-site Trial	Race	Fisher	0.952458	1
Overall Enrollment	Peripheral Vascular Disease	Fisher	1.52E-21	6.22E-19
Overall Enrollment	Antiepileptics	Fisher	0.000705	0.287453
Overall Enrollment	Pneumonia	Fisher	0.00197	0.803666
Overall Enrollment	Female	Fisher	0.348335	1
Overall Enrollment	Osteoarthritis	Fisher	0.634098	1
Overall Enrollment	Urinary Tract Infectious Disease	Fisher	0.461448	1
Overall Enrollment	Viral Hepatitis C	Fisher	1	1
Overall Enrollment	Malignant Tumor Of Urinary Bladder	Fisher	0.467085	1
Overall Enrollment	Primary Malignant Neoplasm Of Prostate	Fisher	1	1
Overall Enrollment	Diabetes Mellitus	Fisher	0.237641	1
Overall Enrollment	Chronic Obstructive Lung Disease	Fisher	0.03708	1
Overall Enrollment	Atrial Fibrillation	Fisher	0.003229	1
Overall Enrollment	Heart Failure	Fisher	0.156308	1
Overall Enrollment	Hypertensive Disorder	Fisher	0.504168	1
Overall Enrollment	Coronary Arteriosclerosis	Fisher	0.003811	1
Overall Enrollment	Gastroesophageal Reflux Disease	Fisher	0.565373	1
Overall Enrollment	Heart Disease	Fisher	0.263693	1
Overall Enrollment	Cerebrovascular Disease	Fisher	0.004355	1
Overall Enrollment	Malignant Lymphoma	Fisher	0.069593	1
Overall Enrollment	Hyperlipidemia	Fisher	0.020105	1
Overall Enrollment	Obesity	Fisher	0.31562	1
Overall Enrollment	Depressive Disorder	Fisher	0.741321	1
Overall Enrollment	Malignant Neoplastic Disease	Fisher	0.554142	1
Overall Enrollment	Venous Thrombosis	Fisher	0.29915	1
Overall Enrollment	Acute Respiratory Disease	Fisher	0.105066	1
Overall Enrollment	Renal Impairment	Fisher	0.57894	1
Overall Enrollment	Hematologic Neoplasm	Fisher	0.627441	1
Overall Enrollment	Lesion Of Liver	Fisher	0.467085	1
Overall Enrollment	Malignant Tumor Of Breast	Fisher	1	1
Overall Enrollment	Visual System Disorder	Fisher	0.942639	1
Overall Enrollment	Ischemic Heart Disease	Fisher	0.199169	1
Overall Enrollment	Chronic Liver Disease	Fisher	0.10817	1
Overall Enrollment	Drugs For Acid Related Disorders	Fisher	0.490595	1
Overall Enrollment	Drugs Used In Diabetes	Fisher	0.003832	1
Overall Enrollment	Antithrombotic Agents	Fisher	0.256029	1
Overall Enrollment	Antineoplastic Agents	Fisher	0.041679	1

© 2021 Rogers JR et al. *JAMA Network Open*.

Trial Characteristic	Covariate Label	Statistical Test	P-value	Adjusted P-value
Overall Enrollment	Diuretics	Fisher	0.930426	1
Overall Enrollment	Beta Blocking Agents	Fisher	0.093952	1
Overall Enrollment	Calcium Channel Blockers	Fisher	0.096786	1
Overall Enrollment	Agents Acting On The Renin-Angiotensin System	Fisher	0.003531	1
Overall Enrollment	Lipid Modifying Agents	Fisher	0.100931	1
Overall Enrollment	Antibacterials For Systemic Use	Fisher	0.033745	1
Overall Enrollment	Drugs For Obstructive Airway Diseases	Fisher	0.008175	1
Overall Enrollment	Immunosuppressants	Fisher	0.009747	1
Overall Enrollment	Antiinflammatory And Antirheumatic Products	Fisher	0.129112	1
Overall Enrollment	Opioids	Fisher	0.04205	1
Overall Enrollment	Psycholeptics	Fisher	0.033461	1
Overall Enrollment	Antidepressants	Fisher	0.040592	1
Overall Enrollment	Age	Fisher	0.10626	1
Overall Enrollment	Ethnicity	Fisher	0.880696	1
Overall Enrollment	Race	Fisher	0.184596	1

© 2021 Rogers JR et al. *JAMA Network Open*.
